# Local and Systemic Immune Responses in Growing Feather Pulps and Blood of Broiler Chickens Elicited by Electron Beam- and Formalin-Killed *Staphylococcus aureus* Vaccines

**DOI:** 10.3390/vaccines14080716

**Published:** 2026-08-20

**Authors:** Ruvindu Perera, Jossie M. Santamaria, Chrysta N. Beck, Gisela F. Erf, Adnan Alrubaye, Palmy Jesudhasan

**Affiliations:** 1 Cell and Molecular Biology Program, University of Arkansas, Fayetteville, AR 72701, USA; rperera@uark.edu; 2 Division of Agriculture, Center of Excellence for Poultry Science, University of Arkansas System, Fayetteville, AR 72701, USA; jmsantam@uark.edu (J.M.S.); gferf@uark.edu (G.F.E.); 3 Department of Poultry Science, Mississippi State University, Mississippi, MS 39762, USA; cnb302@msstate.edu; 4 Poultry Production and Product Safety Research Unit, United States Department of Agriculture-Agricultural Research Service, Fayetteville, AR 72791, USA

**Keywords:** *Staphylococcus*, eBeam, vaccine, leukocytes, broiler chicken

## Abstract

Background: *Staphylococcus aureus* (SA) causes septicemia and arthritis, eventually resulting in substantial economic losses and health hazards in poultry. Lethal electron beam (eBeam) treatment kills bacteria while preserving surface epitopes. Methods: This study compared broiler chicken immune responses (local and systemic) after eBeam-killed (eB) or formalin-killed (FK) SA injections. The vaccine (sham) control was endotoxin-free PBS. This study contained six treatments (trt) with five chickens/trt, with vaccine trts divided into two groups, A and B. Group A received *in ovo* vaccine/sham treatments (phase 1) initially. At 34 d of age, the pulps of growing feathers (GFs) received intradermal (i.d.) injections of the respective trt to elicit booster and primary responses in groups A and B, respectively. Blood was collected to analyze leukocyte populations and plasma SA-specific antibody levels. Additionally, in phase 2, GFs were collected to assess leukocyte presence in GF pulps. Two-way ANOVA was conducted to test the effects of treatment, time, and their interactions, followed by Tukey’s HSD tests at *p* < 0.05 for statistical significance. Results: Early in phase 1, the eB group had increased SA-specific IgM, IgA, and total lymphocyte concentrations compared with FK. In phase 2, FK and eB i.d. vaccines increased total lymphocyte and T cell proportions in GF pulps compared with sham. In blood, the recall eB vaccination increased monocytes, B cells, CD8+ T cells, and total lymphocytes, while the recall FK vaccination increased heterophils (*p* < 0.05). Conclusions: Improved early protection at mucosal surfaces may be reflected by higher plasma levels of SA-specific IgM and IgA following *in ovo* eBeam-killed-SA vaccination. Additionally, both eB-SA and FK-SA vaccines stimulated robust local inflammatory responses dominated by lymphocytes in GF pulps.

## 1. Introduction

*Staphylococcus aureus* is a clinically significant human pathogen widely associated with infective endocarditis and bacteremia, leading to a plethora of complications, including osteoarticular infections, skin and soft tissue infections, pleuropulmonary infections, urinary tract infections, and infections related to medical devices [1]. *S. aureus* (SA) is among the top five pathogens recorded as responsible for over 50% of global deaths caused by bacterial pathogenesis in 2019 [2], with *S. aureus* alone having caused approximately 1 million deaths. Within the United States, *S. aureus* has been shown to cause over 100,000 bloodstream infections per year, leading to virtually 20,000 deaths, while persisting as a major cause of hospital-acquired infections, with roughly 500,000 cases reported annually in the US [3]. Despite the decrease in SA bacteremia-related mortalities over the last three decades, a meta-analysis from 1991 through 2021 emphasizes the urgency of sustained improvement in healthcare to prevent the projected death of 1 in every 4 patients via *S. aureus*-related bacteremia within 3 months [4]. A meta-analysis investigating the prevalence of *S. aureus* across various food sources, as recorded in multiple databases from 2012 through 2022, concluded that meat products accounted for approximately 60% of all SA contaminations, with 22% from chicken and 6% from turkey [5]. Additionally, the discovered SA exhibited resistance mainly to penicillin (61%), with 10% of isolates resistant to quinolones, amphenicols, and rifamycin. Another recent study investigating microbial contamination of raw chicken meat in Serbia recorded *Staphylococcus* spp. contaminations of chicken from all 38 retail stores inspected, of which approximately 90% were *S. aureus* contaminations, where all SA strains were resistant to cloxacillin, amoxicillin, and Augmentin, and a majority were resistant to tetracycline, erythromycin, and chloramphenicol. In contrast, a minority of SA strains showed resistance to gentamycin and cotrimoxazole [6]. Therefore, chicken meat poses a high risk of *Staphylococcus aureus* infections to consumers globally [7], warranting continued surveillance and preventative measures for *Staphylococcus* infections in chickens.

Conversely, *Staphylococcus aureus* has been linked to multiple complications, including septicemia and arthritis in poultry species [8], which adversely impacts animal welfare. Moreover, *S. aureus* is among the most frequently associated bacteria in the pathogenesis of lameness induced by bacterial chondronecrosis with osteomyelitis (BCO) in broiler chickens [9,10,11,12,13,14,15,16,17], leading to severe skeletal infections, joint inflammation, and necrosis of leg bones, reduced mobility, and poor weight gain. BCO lameness has a devastating impact on animal welfare, resulting in approximately 150 million USD in annual revenue losses for the US poultry industry [18] due to the condemnation of diseased birds at the marketing age. The detrimental impacts of *S. aureus* on human and animal health, food safety, animal welfare, and economic revenue are indisputable. Yet, *S. aureus* persists across multiple ecosystems because of its proven resilience and adaptations that evade the immune responses of a wide array of hosts. Namely, *S. aureus* prevents antibody-mediated opsonization and phagocytosis in mammalian hosts by binding the Fc regions of IgG via *Staphylococcus* protein A, thereby compromising humoral immune responses [19]. Furthermore, certain strains possess anti-phagocytic properties [20], including polysaccharide capsules that interfere with complement-mediated phagocytosis by masking the antigens with which host complement factors could bind [21] during opsonization. Therefore, further investigations into the promotion of robust, targeted, and long-lived defense mechanisms against *S. aureus* are pivotal to understanding effective strategies for controlling infections and diseases, thereby alleviating substantial economic burdens and global health concerns.

Vaccines are a critical, sustainable, and highly promising intervention as alternatives to antibiotics in poultry production and pathogen control, actively reducing the need for antimicrobial treatment and lowering the selection pressure for multi-drug-resistant (MDR) bacteria [22,23], especially since the implementation of industry-wide rules by the FDA for the USA, phasing out the use of antibiotics for growth promotion in livestock and poultry [24,25]. This is considered sensible for *S. aureus*, given the MDR properties of frequently isolated strains from chickens, as cited previously [5,6]. Electron beam (eBeam) radiation is an accelerator-generated beam of highly energized electrons that travel nearly at the speed of light, which dissipate energy upon impact with molecules. These collisions cause the energized electrons of impacted atoms to escape orbiting their nuclei, thus ionizing the molecules. This property of ionizing radiation is a well-known means of inactivating bacteria by targeting their DNA and causing irreversible damage. However, a well-optimized eBeam dose preserves the integrity and structure of bacterial surface proteins and epitopes [26,27,28,29,30], which confer antigenicity and immunogenicity in host organisms, thereby eliciting robust and targeted host immune responses against the bacteria. Therefore, the objective of the present study was to investigate the local and systemic immune responses elicited in broiler chickens following exposure to killed *S. aureus* vaccinations, either via exposure to eBeam or formalin. The underlying hypothesis was that the superior epitope-conservation of bacteria by eBeam would prime a highly efficient immune reaction against *S. aureus* in broiler chickens and retain their immune memory against the pathogen to prevent future infections during the production period. The current study employed a dual-window approach [31] to monitor and assess the local and systemic immune responses of broiler chickens elicited by the vaccinations. This approach leverages intradermal (i.d.) injections into multiple growing feather (GF) pulps. Each GF pulp is a separate dermal tissue that acts as an in vivo test tube. Intradermal injection of GF pulps with antigen enables the study of the consequent dynamics of leukocyte populations at the site of injection, i.e., local immune responses in broiler chickens. Therefore, the concurrent sampling of injected GF and peripheral blood in synchrony, before and at several timepoints after the i.d. injections, reveals local (tissue/cellular) and systemic (blood) immune responses of the broilers [32]. The study further compares the primary and booster effects of eBeam- and formalin-killed *S. aureus* immunizations following *in ovo* injections alone (primary response), i.d. injections alone (primary response), or *in ovo* injections followed by i.d. injections (recall/booster response) in different groups of broiler chickens.

This study is the first longitudinal investigation of anti-*Staphylococcus aureus* systemic and local immune responses (within GF pulps) in broiler chickens, including evaluation of primary and recall responses to eBeam- and formalin-killed vaccines. This study was also a foundational analysis leading to the development of an eBeam-killed multi-strain *Staphylococcus* vaccine, which significantly reduced BCO-induced lameness in broiler chickens by more than 50% [17], within a highly stressful BCO-inducing environment under a natural, aerosolized BCO induction model, which was developed by our research group [33]. The outcomes of the current investigation would therefore provide insights into effective anti-*Staphylococcus* immune defenses and mechanisms in poultry and other animals. The developed vaccine would improve animal welfare, significantly reduce carcass condemnation due to BCO lameness, and ensure consumer safety and health. Finally, the outcomes of this study may be leveraged to use eBeam technology to extend vaccine preparation against *S. aureus* for other animal species, and to assess the suitability of eBeam-killed vaccines to control other pathogens and diseases across a multitude of hosts, including humans.

## 2. Materials and Methods

### 2.1. Bacterial Culture and Vaccine Preparation

All protocols for bacterial culture and vaccine preparation were followed as described in Perera et al. (2025) [17]. The same strain of *Staphylococcus aureus* used for the eBeam-killed multi-bacterial anti-BCO vaccine in Perera et al. (2025) [17], a field isolate obtained from BCO-induced clinically lame broilers, was used to prepare the vaccine in the present study. Briefly, *S. aureus* from a 40% glycerol stock was inoculated into 100 mL of Tryptic Soy Broth (TSB, Difco, Becton Dickinson, Franklin Lakes, NJ, USA) using a sterile cotton swab and incubated overnight at 37 °C. The spent medium was removed by centrifugation the following day. The obtained bacterial pellet was resuspended in TSB for further use at 1 × 10^8^ CFU/mL. A portion of the bacterial culture was shipped to the National Center for Electron Beam Research (NCEBR) at Texas A&M University (TAMU), College Station, Texas, in accordance with USDA-APHIS regulations, for exposure to a high-energy electron beam (eBeam) at a dose of 8 kilogray (kGy). Sample packaging for shipment, eBeam treatment, dose verification, and storage at 4 °C upon receipt were performed as previously described [17]. *S. aureus* inactivation with formalin was also similar to that in our previously cited study, using a procedure described by Kaminski et al. (2014) [34]. Briefly, 100 mL of the *S. aureus* culture was mixed with 0.6% formaldehyde (*v*/*v*) (Electron Microscopy Sciences, Hatfield, PA, USA) and incubated for 48 h under constant stirring at 200 RPM (PC-420D Digital Hot Plate Stirrer, Corning Inc., Corning, NY, USA) at room temperature. After formalin treatment, the killed bacterial culture was centrifuged at 1932× *g* for 15 min at 4 °C. After discarding the supernatant, the obtained pellet was resuspended in an equal volume of phosphate-buffered saline (PBS). This step was repeated to ensure complete removal of formalin and the purity of the killed bacterial culture. The final solution, resuspended in TSB, was stored at 4 °C for further use. Finally, the eBeam- and formalin-treated *S. aureus* were centrifuged separately under the above conditions on the day of vaccination, resuspended in Dulbecco’s endotoxin-free PBS (EF-PBS; MilliporeSigma, Burlington, MA, USA), and diluted to a final concentration of 1 × 10^7^ CFU/mL. The eBeam- and formalin-killed bacterial cultures resuspended in EF-PBS were used as the eBeam- and formalin-killed whole-cell vaccines to vaccinate the birds, as outlined later. Adjuvants were not used, as both vaccines present killed whole-cell bacteria to the hosts’ immune systems; these bacteria contain a multitude of epitopes and are therefore self-immunogenic.

### 2.2. Confirmation of Bacterial Inactivation

As previously cited [17], complete bacterial inactivation was confirmed before preparing the vaccine solution. Both eBeam- and formalin-killed *S. aureus* cultures at 1 × 10^8^ CFU/mL in TSB were serially diluted in PBS and spread-plated onto mannitol salt agar (MSA) plates (Difco, Becton Dickinson, Franklin Lakes, NJ, USA). They were incubated overnight at 37 °C and observed for a week at room temperature for signs of bacterial resuscitation. Moreover, 100 µL from each differently killed bacterial culture was inoculated into freshly prepared TSB in duplicate. One replicate was incubated overnight at 37 °C, while the other was incubated for the same duration at room temperature. Along with the MSA plates, these inoculum tubes were observed at room temperature until broilers were vaccinated and were observed for 3 weeks post-inactivation for any signs of bacterial growth/resuscitation.

### 2.3. Experimental Animals

All procedures and protocols were approved by the University of Arkansas, Division of Agriculture, Institutional Animal Care and Use Committee (IACUC), under Protocol #23016. A total of two hundred and ten (210) fertilized Cobb500 broiler eggs were incubated at 99.6 °F and 85% RH as per IACUC regulations. This number was requested to account for damage during transportation and losses due to poor viability and/or spoilage. Eggs were observed on the day of placement in the incubator (day 0) and on days 10 and 18 of incubation to remove any eggs that were damaged, spoiled, not fertilized, or contained non-viable embryos before vaccine administration. The embryos were *in ovo* vaccinated with 100 µL/egg of either 1 × 10^7^ CFU/mL eBeam-killed *S. aureus* (eBeam vaccine), 1 × 10^7^ CFU/mL formalin-killed *S. aureus* (formalin vaccine), or vehicle (EF-PBS; sham vaccine) as detailed previously [17]. Following vaccine administration, the eggs from each treatment group were placed separately in hatching trays and incubated at 98 °F and 85% RH until hatching. On the day of hatch, a total of one hundred and twenty-six (126) chicks were distributed among six (6) litter-flooring pens of 6ft × 7ft, according to the experimental design, allowing at least 172.8 in^2^ floor space/bird, well exceeding the IACUC-approved stocking density for broilers until the conclusion of the experiment at sixty-three (63) days post-hatch. The broilers were raised under biosecurity conditions, with temperature and lighting controlled in accordance with IACUC standards at the UADA Poultry Research Farm. Water and feed that met or exceeded nutritional requirements were provided ad libitum. All damaged, spoiled, and non-viable eggs at each point of observation, as well as unhatched eggs, were appropriately discarded in accordance with regulatory standards. Additionally, hatched birds, as well as any remaining birds at the end of the experiment, were euthanized.

### 2.4. Experimental Design

The birds were allocated into 2 main treatment groups: group A with 105 birds and group B with 21 birds. Group A broilers received either eBeam (A-EB), formalin (A-FK), or sham (A-Sham) vaccines *in ovo* (35 broilers per treatment), and were raised in separate litter-flooring pens (Table 1). All birds of group B received the sham vaccine *in ovo* and were raised in 3 separate litter-flooring pens, with each pen housing 7 birds. On day 16 post-hatch, 22 breast feathers per bird (11 per side of the breast) were removed and allowed to regenerate for 19 days to obtain uniform growing feathers (GFs) for intradermal vaccination, as per the protocol established by Dr. Gisela Erf [31,35,36]. Adopting previously cited protocols [35,36], on day 35, all birds received intradermal (i.d.) GF-pulp injections of vaccine (10 μL per GF; 22 GFs/bird) as detailed below. All birds of group A received i.d. GF-pulp injections of the same vaccine that they had received *in ovo*, and seven birds in each of group B received primary i.d. GF-pulp injections of either one of the eBeam (B-EB), formalin (B-FK), or sham (B-Sham) vaccines. Table 1 below elaborates on the treatment design. The pre-GF pulp i.d. injection period (day 0-day 34) was considered phase 1 (P1), and the post-GF pulp i.d. injection phase was considered phase 2 (P2) for convenience.

### 2.5. Sample Collection

**Figure 1:**
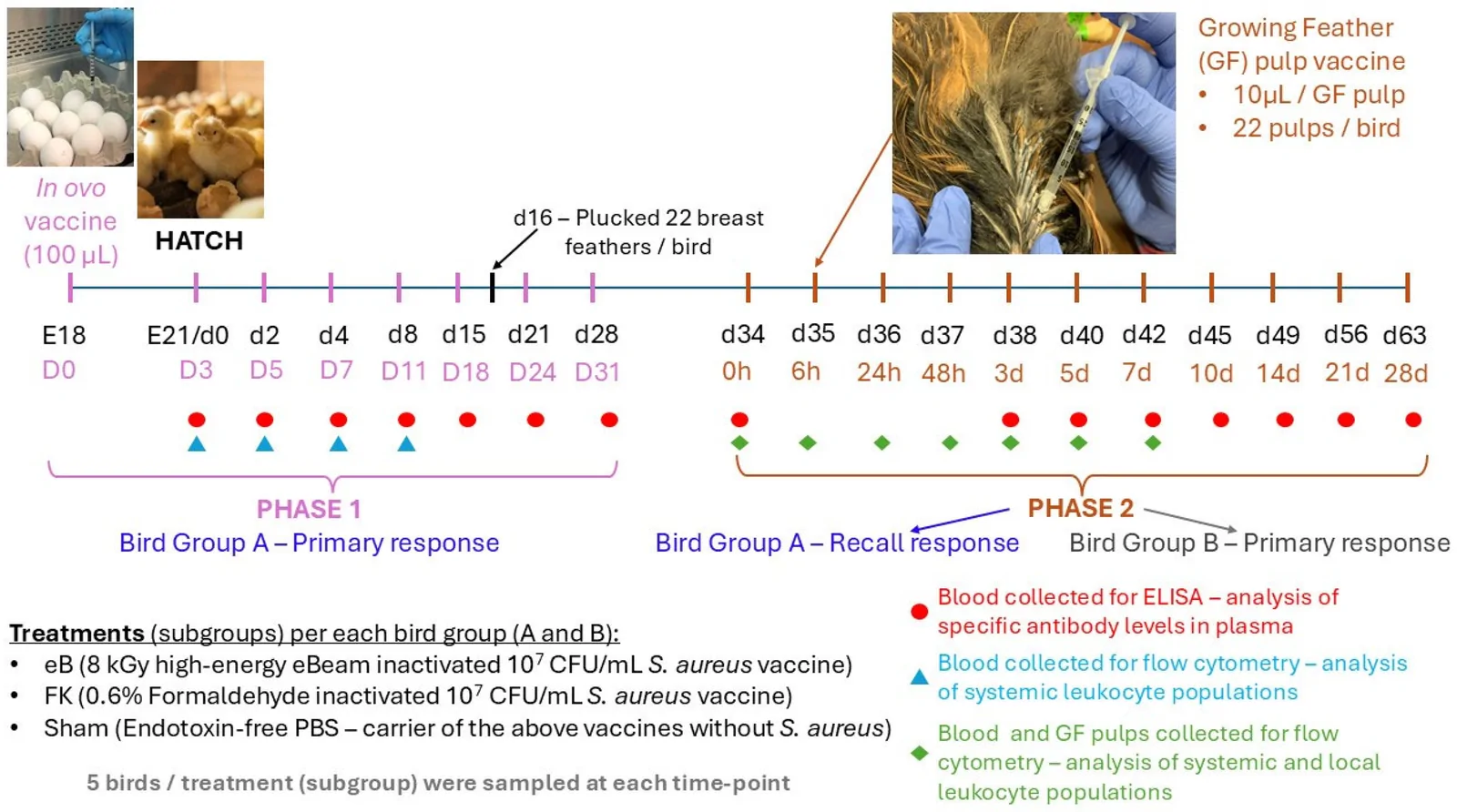
Timeline of the experiment. Days in black: E18–E21—age of embryo; d0–d63—age of birds post-hatch; days in pink: D0–D31—days post *in ovo* vaccination in phase 1; hours and days in orange: 0 h–28d—days post intradermal vaccination in phase 2.

During the first phase of the study, blood was collected from 5 birds/treatment on days 3 through 31 (D3, D5, D7, D11, D18, D24, and D31) post *in ovo* vaccination (which corresponds to days 0, 2, 4, 8, 15, 21, and 28 post-hatch) from the birds of group A. Birds were sacrificed, and blood was collected via cardiac puncture from randomly selected birds for the 1st 4 sampling timepoints, as their small size made venipuncture and collection of the required blood volume difficult. For the remainder of P1 (15, 21, and 28 days of age), 1–1.5 mL of heparinized blood was collected from the brachial vein. Heparinized 3 mL syringes with 25G 1-inch needles were used for all blood collections. The collected blood samples were centrifuged at 10,000× *g* for 3 min at room temperature, and plasma samples were stored at −80 °C until *S. aureus* (SA)-specific antibody levels were determined. During P2, heparinized blood was collected before (0 h) and at 6 h, 24 h, 48 h, 3 d, 5 d, 7 d, 10 d, 14 d, 21 d, and 28 d post i.d. GF-pulp injections from 5 birds/treatment, and plasma samples were stored similarly for the analysis of SA-specific antibody levels. A portion of the heparinized blood collected from each bird (5/treatment) on D3 through D11 in P1, and 0 h through 7 d in P2, respectively, was processed for same-day analysis of leukocyte population profiles before centrifugation and plasma extraction. Furthermore, one GF pulp/bird was collected from 5 birds/treatment before (0 h), and at 6 h, 24 h, 48 h, 3 d, 5 d, and 7 d post i.d. injections. Collected GF pulps were placed in ice-cold PBS and kept on ice until processing for same-day pulp leukocyte population analysis. Figure 1 below shows the experiment timeline and a summary of the sampling schedule.

### 2.6. Quantification of S. Aureus-Specific Immunoglobulins in Blood Plasma

Modified recent adaptations [35,36] of previously established protocols [37,38,39] for enzyme-linked immunosorbent assay (ELISA) were used for comparative evaluation of the relative levels of SA-specific antibody isotypes: IgM, IgY (avian IgG counterpart), and IgA in the plasma of vaccinated broilers. The detailed protocol, reagents, antibodies, and equipment described by Perera et al. (2025) [17] were utilized with optimizations to the antibody and plasma dilutions. Briefly, a 1 × 10^7^ CFU/mL culture of *Staphylococcus aureus,* suspended in coating buffer, was used to coat the 96-well flat-bottom plates for the assay as previously detailed. The coated plates were stored at 4 °C until further use. The plates were allotted based on the isotype of antibody being evaluated, and the non-specific binding sites of the wells were blocked with blocking solution, followed by washing the ELISA plates as previously detailed. The frozen plasma samples from vaccinated broilers were thawed and diluted in diluent according to previously determined pretest titers. P1 plasma samples were diluted to 1/25, 1/100, and 1/25, and P2 plasma samples were diluted to 1/400, 1/100, and 1/50 for the detection of IgM, IgY, and IgA, respectively. The detection antibodies IgM, IgY, and IgA (HRP-conjugated, goat anti-chicken antibodies) were diluted to 1/5000, 1/10,000, and 1/2500, respectively, with the same diluent. Nine doubling dilutions of sample pools (plasma samples from the eB and FK treatments, prepared separately for each phase) were included in all assays as positive controls and to generate a standard curve. The diluted plasma samples were added to the plates in triplicate, followed by incubation, washing, addition of the detection antibodies, washing, and addition of the 3,3′,5,5′-tetramethylbenzidine substrate solution (Thermo Fisher, Waltham, MA, USA). Finally, the reaction was stopped with 2 M sulfuric acid after 15 min of incubation at 37 °C, and the absorbance at 450 nm was measured. The absorbance units (AU) of diluted sample pools were used to generate a standard curve for each plate. The resulting line equation was used to adjust the relative AU levels of each ELISA plate.

### 2.7. Preparation and Immunofluorescent Staining of GF Pulp and Blood Cell Suspensions, and Cell Population Analysis by Flow Cytometry

GF-pulp cells were prepared for flow cytometry analysis based on adaptations of previously established protocols [32,36,40,41]. Briefly, the GF pulps were removed from their feather sheaths using scalpels and forceps at each timepoint and were separately placed in microcentrifuge tubes with 0.5 mL of 0.1% collagenase/dispase (Collagenase Type IV, Life Technologies, Carlsbad, CA, USA; Dispase II, Sigma-Aldrich, St. Louis, MO, USA), and incubated at 37 °C for 15 min. The incubated suspensions were passed through 60 µm nylon mesh filters with ice-cold PBS using the blunt ends of 3 mL syringe plungers to generate single-cell pulp suspensions. The obtained GF-pulp suspensions were centrifuged in 15 mL centrifuge tubes to obtain pellets; the supernatants were discarded, and the pellets were resuspended in ice-cold PBS. The washing steps were repeated, and GF pulp pellets were resuspended in 0.25 mL/pellet of PBS+ (PBS, 0.1% Sodium Azide, and 1% bovine serum albumin (BSA; VWR, Radnor, PA, USA) and kept on ice until fluorescent staining.

To prepare whole-blood cell suspensions, 20 µL of each blood sample was diluted 1:50 in 980 µL of PBS+. For immunofluorescent (IF) staining, mouse anti-chicken (mac) monoclonal antibodies (Southern Biotechnology Associates, Inc., Birmingham, AL, USA), specific for chicken leukocyte cell surface markers, and fluorescently labeled with different fluorophores, were used in a 3-color direct IF staining procedure based on published protocols [32,36,40,42] (Table 2). For IF staining of pulp cell suspensions, 50 µL of GF-pulp cell suspensions were incubated with 50 µL of antibody cocktails for 30 min at 4 °C, washed with PBS+ twice, and resuspended in a final volume of 200 µL of PBS+ for flow cytometry analysis. For IF of whole blood cell suspensions, 50 µL of blood cell suspensions were incubated with 50 µL of antibody cocktails (blood dilution 1:100) for 45 min at 4 °C, then diluted in 300 µL of PBS+ to a 1:400 dilution before flow cytometry analysis. Staining controls included appropriate isotype controls and single-stained samples. Cell population analyses were conducted using the BD C6-Plus Accuri flow cytometer (Becton Dickinson, San Jose, CA, USA), followed by analysis of the acquired data using FlowJo software v10.10 (FlowJo, LLC, Ashland, OR, USA), in accordance with previously established protocols [43]. GF-pulp cell populations were expressed as a percentage (%) of total pulp cells. Concentrations of various leukocyte populations in the blood (10^3^ cells/μL) were calculated by multiplying the percentage of a cell type in the blood cell suspension by the concentration of cells in the suspension and the 400× dilution factor and dividing the result by 100.

### 2.8. Statistical Analysis

All statistical analyses were performed using JMP Pro software version 18.0.2 (JMP Statistical Discovery LLC, Cary, NC, USA), and all data were visualized using GraphPad Prism software version 10.2.0 (GraphPad Software, San Diego, CA, USA). Five broilers were sampled per treatment at each data collection timepoint, and the experimental units were the individual broilers. Two-way ANOVA followed by Tukey’s multiple-mean comparison tests were used to analyze the effects of time, treatment, and time-by-treatment interactions on antibody levels and concentrations of blood leukocyte populations for phase 1. For phase 2, antibody-level and leukocyte population data were analyzed using two-way repeated-measures ANOVA, because the same bird was tracked in each treatment for antibody and leukocyte responses in the blood. However, traditional two-way ANOVA was used to analyze leukocyte populations in GF pulps, as each pulp is a separate experimental unit. During P2, recall and primary responses for groups A and B were analyzed separately. No further adjustments were made following the post hoc tests. Data were presented as means ± standard errors of the means (SEMs). Statistical significance was considered at *p* values ≤ 0.05.

## 3. Results

### 3.1. Confirmation of Bacterial Inactivation Prior to Vaccination

After spread plating eBeam- and formalin-killed cultures onto MSA, no colonies were observed from the day of plating through 7 days post-plating, with overnight incubation at 37 °C followed by room-temperature incubation for the remainder of the period. Similarly, none of the 50 mL tubes containing freshly prepared TSB inoculated with the eBeam- and formalin-killed cultures showed turbidity (bacterial growth) for 3 weeks post-inoculation, whether incubated exclusively at room temperature or incubated at 37 °C overnight, then at room temperature for the remainder of the period. This confirmed the complete inactivation/reproductive death of both eBeam- and formalin-killed *S. aureus*.

### 3.2. Staphylococcus Aureus-Specific IgM, IgY, and IgA Antibody Levels in the Plasma of Birds After In Ovo Injections

**Figure 2:**
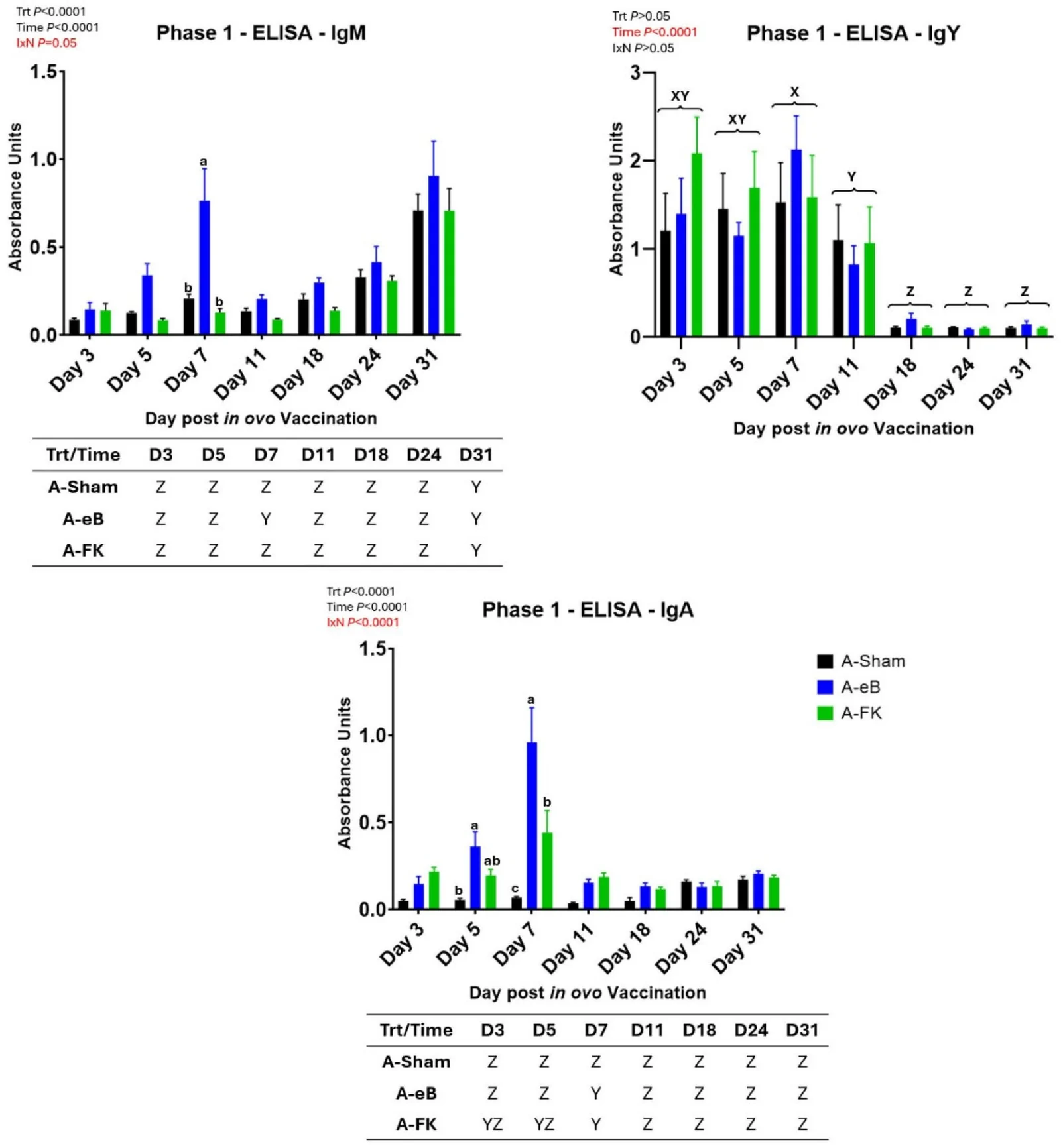
*Staphylococcus aureus* (SA)-specific IgM, IgY, and IgA levels in the blood of newly hatched broiler chickens on days 3, 5, 7, 11, 18, 24, and 31 post *in ovo* vaccination (PV) on embryonic day 18 of group A broilers with eBeam-killed SA (A-eB), formalin-killed SA (A-FK), or vehicle (A-Sham) vaccines during phase 1 of the experiment. Up to 1.5 mL of heparinized blood was collected from 5 birds per treatment at each timepoint via cardiac puncture for days 3–11 PV, followed by venipuncture for days 18–31 PV from the brachial wing vein. ELISA was used to quantify the relative levels of SA-specific antibodies. Two-way ANOVA was used to analyze the effects of treatment (trt), time, and their interaction (trt × time; IxN), and results are presented as mean absorbance units ± SEM. When interactions were significant (*p* ≤ 0.05), Y, Z: timepoints are significantly different within the considered trt unless they share the same letter, and a–c: trts are significantly different within the considered timepoint unless they share the same letter. When interactions were not significant (*p* > 0.05), X–Z: statistical differences between the main effects of timepoints are denoted by different letters.

The production of SA-specific antibodies was evaluated in group A birds during P1 of the experiment, following *in ovo* vaccination with either eBeam-killed SA, formalin-killed SA, or sham vaccines, on days 3, 5, 7, 11, 18, 24, and 31 post-vaccination. Figure 2 below shows changes in the antibody levels over time, reflecting a primary antibody response to the *in ovo* vaccinations.

Following *in ovo* vaccinations of 18-day-old embryos, interactions of treatment and time were marginally significant (*p* = 0.05) for plasma IgM levels. SA-specific IgM levels in birds injected with the FK and sham vaccines remained low from D3 through D24 PV, then increased significantly on D31 PV. The average plasma IgM levels for the birds in the A-eB group followed a similar trend, except for a significant increase in IgM levels on D7 PV, at which time A-eB levels were greater than those in the A-FK and A-Sham groups. All 3 treatment groups had the highest IgM levels on D31 PV, which did not differ significantly from each other (Figure 2). For circulating levels of SA-specific IgY antibodies, the main effects of treatment and the treatment-by-time interaction were not statistically significant. However, the main effects of time were significant (*p* < 0.0001), whereby IgY levels were similarly high for all treatments on D3, 5, and 7 PV, followed by a decline (*p* < 0.05) on D11 PV, reaching the lowest (*p* < 0.05) levels by D18 PV and remaining at this level through D31 PV. The highest plasma IgY levels were recorded on D7 PV (Figure 2). Circulating SA-specific IgA levels post *in ovo* vaccination showed significant (*p* < 0.0001) trt × time interaction effects. The IgA levels of the A-Sham group remained near baseline across all timepoints, while both the A-eB and A-FK vaccine groups showed a gradual increase in IgA from D3 to the highest levels at D7, followed by a return to baseline from D11 to D31. On D5, IgA levels in A-eB were higher (*p* < 0.0001) than in A-Sham, with A-FK levels between those in the A-Sham and A-FK groups. On D7, A-eB had the highest IgA levels (*p* < 0.0001), followed by A-FK, with both groups having higher than the baseline levels in the A-Sham group (Figure 2). In summary, A-eB had significantly elevated SA-specific IgM and IgA levels compared to the other groups by D7 PV. IgY behaved similarly across all treatments, possibly indicative of maternal immunity.

### 3.3. Leukocyte Concentration Profiles in the Blood of In Ovo Vaccinated Broiler Chickens

**Figure 3:**
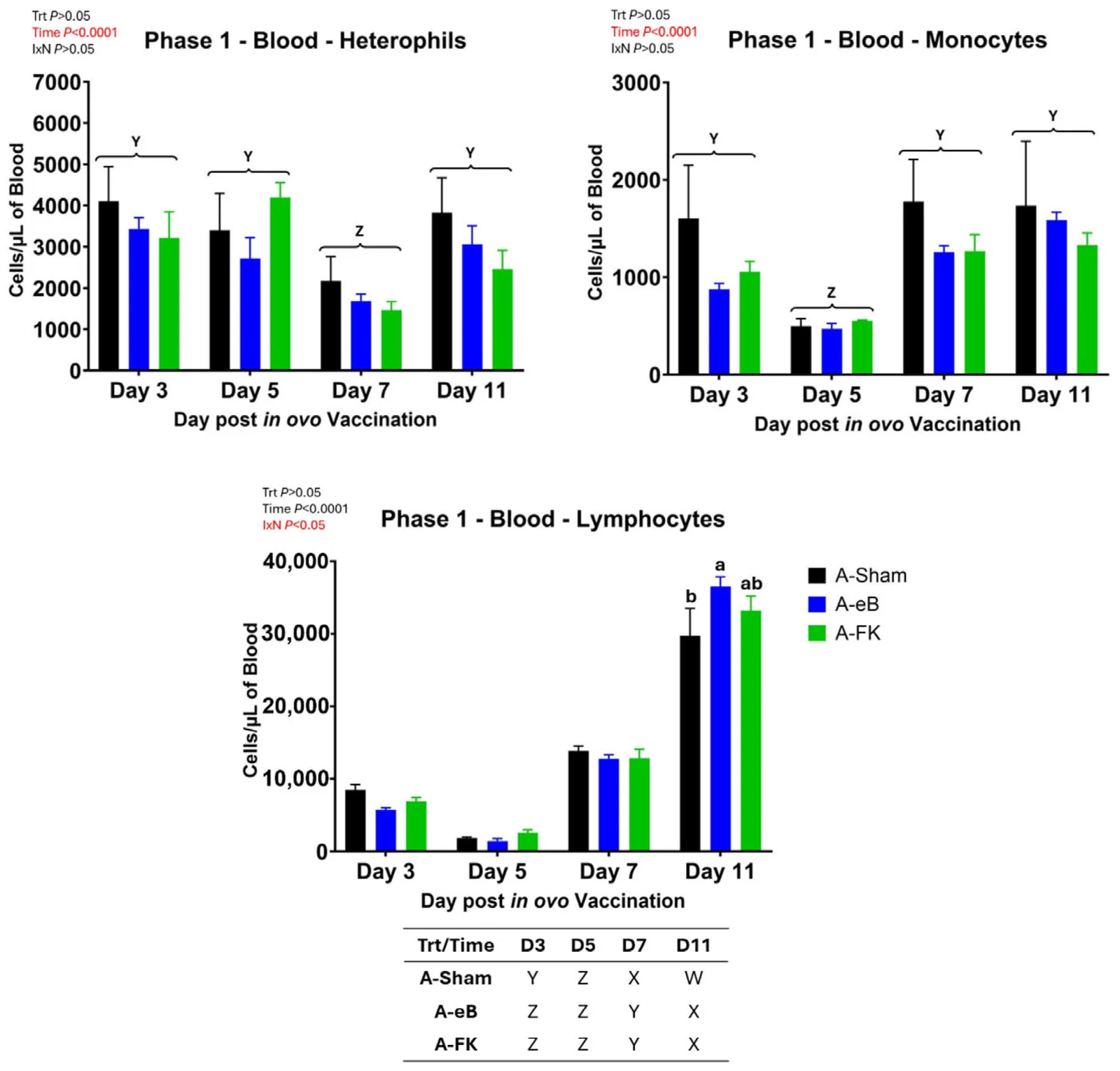
Heterophil-, monocyte-, and total lymphocyte concentrations in the blood of newly hatched broiler chickens at 3, 5, 7, and 11 days post *in ovo* vaccination (PV) on embryonic day 18 of group A broilers with eBeam-killed *Staphylococcus aureus* (A-eB), formalin-killed SA (A-FK), or vehicle (A-Sham) vaccines during phase 1 of the experiment. Up to 1.5 mL of heparinized blood was collected via cardiac puncture from 5 birds per treatment at each timepoint. Leukocyte populations in whole blood were identified separately by immunofluorescent staining with fluorescence-conjugated, chicken leukocyte-specific mouse monoclonal antibodies, followed by fluorescence-based flow cytometry. Data were analyzed by two-way ANOVA (treatment (trt), time, and their interaction (IxN)) and are presented as the mean concentrations (cells/µL of blood) of the leukocyte populations ± SEM. When interactions are significant (*p* ≤ 0.05), W–Z: timepoints that do not share the same letter are statistically different within the considered trt, and a, b: trts that do not share the same letter are statistically different within the considered timepoint. When interactions are not significant (*p* > 0.05), X–Z: main effects of timepoints that do not share the same letter are statistically different.

Leukocyte profiles of newly hatched chicks were evaluated post *in ovo* vaccination with SA-eB, SA-FK, or sham vaccines (group A broilers) on D3, 5, 7, and 11 PV by immunofluorescent staining followed by flow cytometry of blood samples. There were no main effects of trt or trt × time interactions for both heterophil and monocyte concentrations (Figure 3). However, the main effects of time were significant (*p* < 0.0001) for both heterophil and monocyte concentrations. Independent of treatment, heterophil concentrations were similar on D3, 5, and 11 PV and higher than on D7 PV. In contrast, monocyte concentrations did not differ on D3, 7, and 11 PV but were higher than on D5 PV. Contrary to the above trends, there were significant (*p* < 0.05) trt × time interaction effects on total lymphocyte concentrations. In general, all three treatment groups had the lowest total lymphocyte concentrations on D3 and 5 PV, increased (*p* < 0.05) on D7, and then rose to the highest (*p* < 0.05) concentrations on D11 PV. The average total lymphocyte concentrations between treatment groups at each timepoint were not significantly different from D3 through D7 PV. However, on D11, the lymphocyte concentration for the A-eB group was higher (*p* < 0.05) than that of the A-Sham group. In contrast, the lymphocyte concentration in the A-FK group was intermediate and not different from either group (Figure 3).

**Figure 4:**
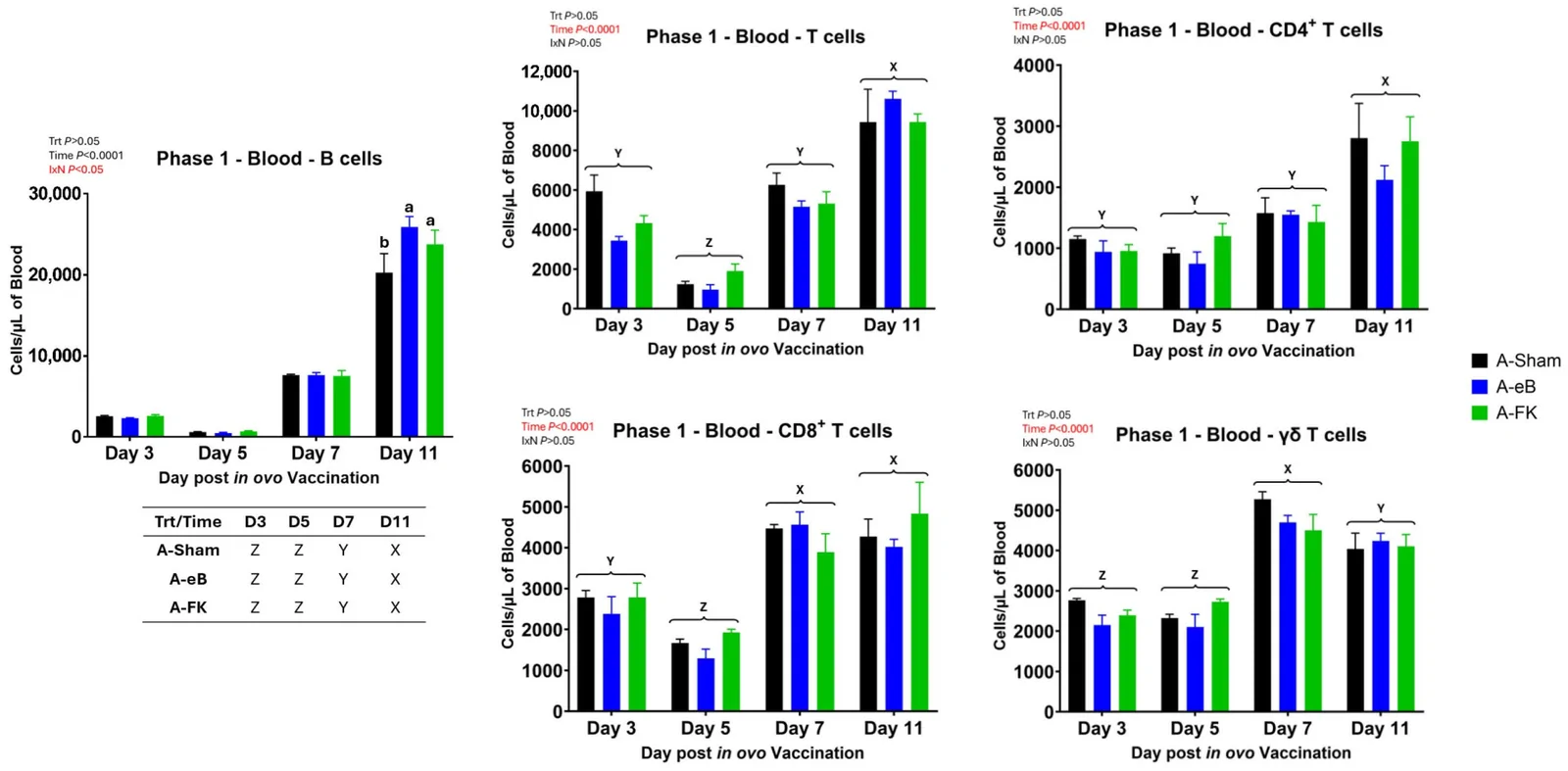
Concentrations of B- and T- lymphocyte populations in the blood of newly hatched broiler chickens on days 3, 5, 7, and 11 after *in ovo* vaccination (PV) on embryonic day 18 with eBeam-killed *Staphylococcus aureus* (A-eB), formalin-killed SA (A-FK), or vehicle (A-Sham) vaccines during phase 1 of the experiment. Up to 1.5 mL of heparinized blood was collected via cardiac puncture from 5 birds per treatment at each timepoint. Leukocyte populations in whole blood were identified by immunofluorescent staining with fluorescence-conjugated, chicken leukocyte-specific mouse monoclonal antibodies, followed by fluorescence-based flow cytometry. Data were analyzed using two-way ANOVA (treatment (trt), time, and their interaction (IxN)) and are presented as mean concentrations (cells/µL of blood) of leukocyte populations ± SEM. When interactions are significant (*p* ≤ 0.05), X–Z: timepoints that do not share the same letter are statistically different within the considered trt, and a, b: trts that do not share the same letter are statistically different within the considered timepoint. When interactions are not significant (*p* > 0.05), X–Z: main effects of timepoints that do not share the same letter are statistically different.

Among the blood lymphocyte subpopulations, interaction effects between treatment and time were observed in circulating B lymphocytes (Figure 4). The B cell concentrations across all three treatment groups followed the same trend: they were lowest on D3 and D5 PV, then increased significantly on D7, reaching maximal levels on D11 PV. On D11, B cell concentrations were greater (*p* < 0.05) in A-eB and A-FK than in A-Sham broilers and did not differ between them. For the concentrations of total T cells and the T cell subsets, there were only main effects of time (*p* < 0.001) and no effects of treatment or trt × time interactions (Figure 4). Concentrations of total T cells dropped to the lowest levels on D5 PV, then increased again to D3 levels on D7, before reaching the highest concentrations (*p* < 0.05) on D11 PV. Independent of treatment, CD4+ T cell concentrations did not change from D3 through D7 but increased on D11 PV (*p* < 0.05). Similar to total T cell concentrations, CD8+ T cells dropped (*p* < 0.05) to their lowest levels from D3 to D5 PV, then increased (*p* < 0.05) to levels above D5 concentrations on D7 and remained at this level on D11. γδ T cell concentrations did not change from D3 to D5, then increased (*p* < 0.05) to peak levels on D7 before dropping to lower but still above (*p* < 0.05) D3 and D5 concentrations on D11. At any given timepoint, there were no significant differences in concentrations between any of the treatments, A-eB, A-FK, or A-Sham, for total T cells as well as T cell subpopulations. In summary, heterophils, monocytes, and T cells showed similar dynamics across treatments, but A-eB and A-FK had elevated B cell concentrations compared with A-Sham by D11 PV.

### 3.4. Changes in Leukocyte Profiles in Growing Feather Pulps of Broiler Chickens in Response to Primary or Secondary Intradermal Pulp Vaccinations

**Figure 5:**
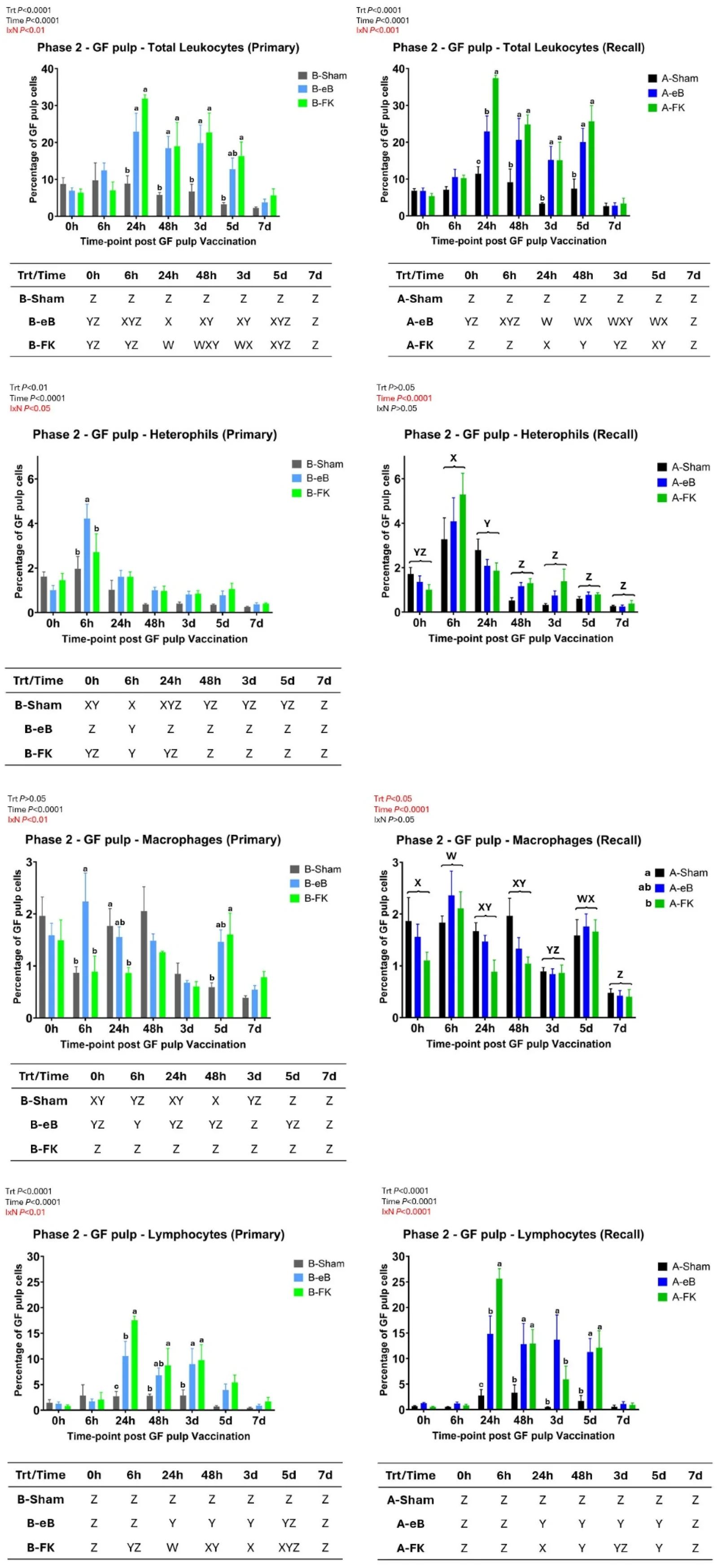
Proportions of total leukocytes, heterophils, macrophages, and total lymphocytes in the pulps of growing feathers of 5-week-old broiler chickens before (0 h) and at 6 h, 24 h, 48 h, 3 d, 5 d, and 7 d after intradermal vaccination (PV) with either eBeam-killed *Staphylococcus aureus* (A-eB, B-eB), formalin-killed SA (A-FK, B-FK), or vehicle (A-Sham, B-Sham) vaccines. The vaccines were administered by intradermal (i.d.) injection of growing feather (GF) pulps (10 μL/GF; 22 GF/bird). The data shown depict the dynamics of primary- and recall-leukocyte infiltration responses in group B broilers, which were not *in ovo* vaccinated, and in group A broilers, which received the same vaccines *in ovo* on embryonic day 18 during phase 1, respectively. Leukocyte populations in GF-pulp cell suspensions were identified by immunofluorescent staining with fluorescence-conjugated, chicken leukocyte-specific mouse monoclonal antibodies, followed by fluorescence-based flow cytometry. Leukocyte data were analyzed using two-way ANOVA (treatment (trt), time, and their interaction (IxN)) and are presented as mean percentage of total pulp cells (% pulp cells) ± SEM. When IxN were significant (*p* ≤ 0.05), W–Z: for each trt, timepoints that do not share the same letter are different, and a–c: trt that do not share the same letter within a timepoint are different. When IxN were not significant (*p* > 0.05), W–Z or a, b: main effects of timepoint or trt that do not share the same letter are different.

The GF pulps are dermal tissues that provide a window into immune cell recruitment, thereby allowing visualization of local inflammatory responses at intradermal vaccine injection sites. Analysis of total leukocyte levels (% pulp cells) in GF pulps following the primary (group B) and recall (group A) i.d. vaccine administration revealed trt × time interaction effects at *p* < 0.01 and *p* < 0.001, respectively (Figure 5). In both responses, leukocyte proportions in GF pulps did not change over time in sham-vaccinated broilers post-i.d. vaccination (PV). In both responses, leukocyte levels in eB and FK broilers increased sharply at 24 h PV and remained elevated (*p* < 0.05) through 5 d, before returning to pre-injection levels at 7 d PV. For the primary response, both B-eB and B-FK groups had similar total leukocyte percentages from 24 h through 3 d PV, which were higher (*p* < 0.05) than those of the B-Sham group. At 5 d PV, the B-FK broilers had significantly higher levels of leukocytes in GF pulps than the B-Sham group, while those of the B-eB group were between B-Sham and B-FK levels and not different (*p* > 0.05) from either group, although numerically closer to the B-FK group. During the recall response, both A-eB and A-FK maintained higher (*p* < 0.05) total leukocyte proportions in GF pulps than the A-Sham group from 24 h through 5 d PV. Leukocyte presence in GF pulps of the A-eB and A-FK groups was similar across the above timepoints, except at 24 h PV, where A-FK had higher (*p* < 0.05) total leukocyte percentages than A-eB.

For heterophils, there was a trt by time interaction effect for the primary response (Figure 5). For all treatment groups, heterophil proportions peaked at 6 h PV and then returned to near- or below-pre-injection levels thereafter. Heterophil levels across all vaccine groups were similar throughout most of the primary response, except at 6 h PV, when B-eB had higher (*p* < 0.05) mean heterophil percentages than both B-FK and B-Sham, which were not significantly different from each other (Figure 5). For the recall response, there was a main effect of time (*p* < 0.0001), with all treatments depicting an increase in heterophil presence to peak levels at 6 h, followed by a drop (*p* < 0.05) at 24 h, and a further decrease to pre-injection levels at 48 h through 7 d PV. The main effects of treatments were not significant for the recall response.

Considering macrophage proportions (Figure 5) in the GF pulps, there were also trt × time interaction effects (*p* < 0.01) during the primary response but not during the recall response. In the primary response, macrophage levels did not change over time in B-FK. In B-eB, macrophage levels slightly increased at 6 h PV and dropped below this level on 3 d and 7d, while in B-Sham, levels dropped at 6 h, then gradually increased to maximal levels at 48 h before dropping (*p* < 0.05) to below pre-injection levels from 3 d to 7 d. Analysis of treatment differences at various timepoints revealed differences (*p* < 0.05) in macrophage levels: at 6 h PV, when B-eB levels were greater than in B-FK and B-Sham, which were not different from each other; at 24 h PV, when B-Sham levels were greater (*p* < 0.05) than B-FK levels and B-eB levels were intermediate and not different from either B-Sham or B-FK; and, on 5 d PV, when B-FK had greater macrophage presence than B-Sham, and levels in B-eB were between those of the B-FK and B-Sham groups. For the recall response, there were main effects of treatment (*p* < 0.05) and time (*p* < 0.0001). All treatments (A-eB, A-FK, A-Sham) showed an increase in macrophage infiltration from 0 h to 6 h PV, followed by a gradual decrease to levels below pre-injection at 7 d PV. A-Sham had higher (*p* < 0.05) macrophage levels throughout compared to the A-FK group, while macrophage levels in the A-eB group were between those of the A-FK and A-Sham groups and not different (*p* > 0.05) from either group.

The percentage of total lymphocytes (Figure 5) showed significant trt × time interaction effects during the primary (*p* < 0.01) and recall (*p* < 0.0001) responses to the i.d. GF-pulp vaccinations. Total lymphocyte proportions in sham-vaccinated birds did not change over time or across responses. During the primary response, the proportions of total lymphocytes in the B-eB and B-FK groups increased (*p* < 0.05) from 0 h to 24 h to maximal levels, remained at (B-eB) or just below (B-FK) this level at 48 h and 3 d PV, then decreased (*p* < 0.05) to pre-injection levels at 7 d PV. At 24 h and 3 d, the B-FK group had higher (*p* < 0.01) lymphocyte levels in GF pulps than the B-eB group, while lymphocyte proportions in the B-Sham group were lower (*p* < 0.05) than in the B-eB and B-FK groups. At 48 h PV, the lymphocyte proportions in the B-FK group were greater (*p* < 0.05) than in the B-Sham group, and those in the B-eB group were between and not different (*p* > 0.05) from B-FK and B-Sham. During the recall response, the lymphocyte proportions in GF pulps of the sham group did not change over time (*p* > 0.05), whereas levels increased to maximal levels at 24 h PV in both the A-eB and A-FK groups. While in the A-eB group maximal lymphocyte proportions were maintained from 24 h through 5 d PV before returning to pre-injection levels at 7 d PV, those of the A-FK group dropped (*p* < 0.05) to lower levels at 48 h and remained near this level through 5 d PV before returning to pre-injection levels at 7 d PV. Total lymphocyte levels in the A-eB and A-FK groups were higher (*p* < 0.05) than in the A-Sham group from 24 h to 5 d PV. Moreover, lymphocyte levels were higher (*p* < 0.05) in A-FK than in A-eB GF pulps at 24 h PV, not different at 48 h and 5 d PV, and higher in A-eB than in A-FK at 3 d PV.

**Figure 6:**
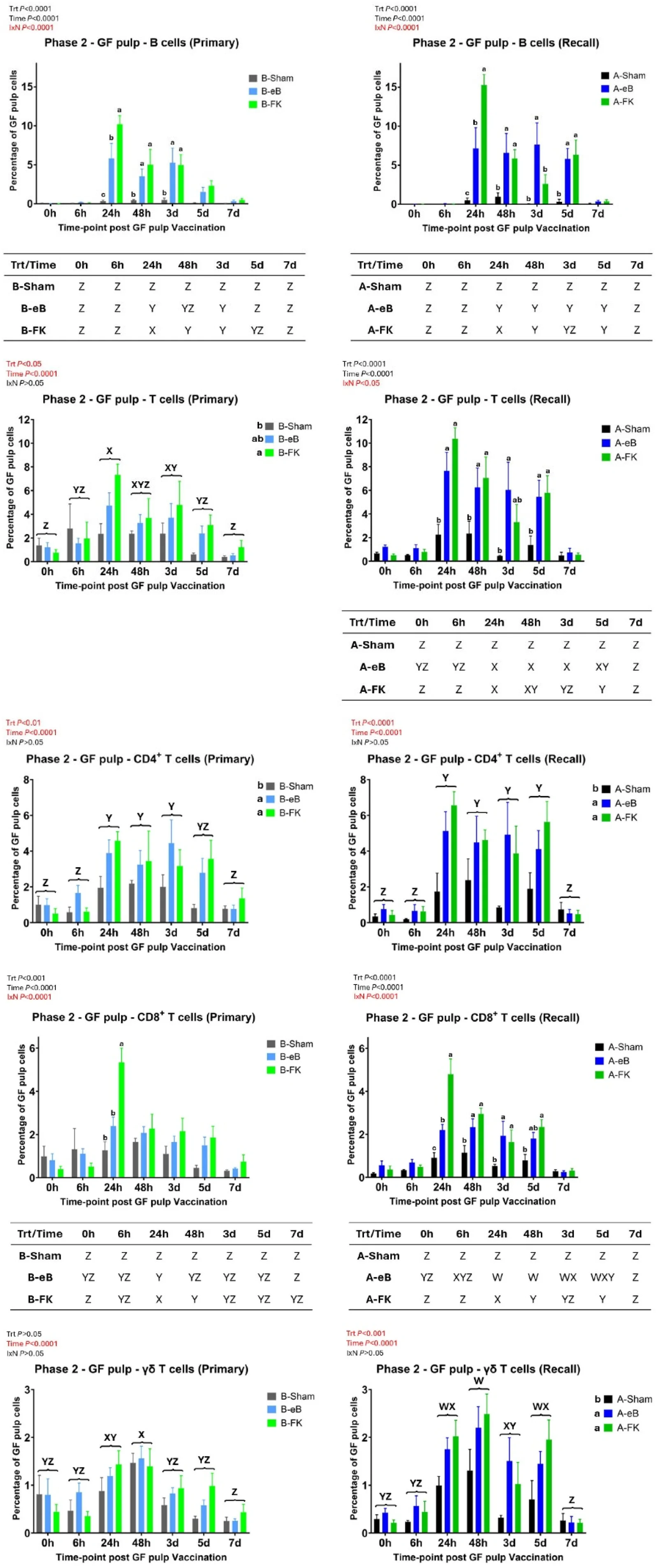
Proportions of lymphocyte populations (B cells, total T cells, CD4+ T cells, CD8+ T cells and γδ T cells) in the pulps of growing feathers of 5-week-old broiler chickens before (0 h) and at 6 h, 24 h, 48 h, 3 d, 5 d, and 7 d after intradermal vaccination (PV) with either eBeam-killed *Staphylococcus aureus* (A-eB, B-eB), formalin-killed SA (A-FK, B-FK), or vehicle (A-Sham, B-Sham) vaccines. The vaccines were administered by intradermal (i.d.) injection of growing feather (GF) pulps (10 μL/GF; 22 GF/bird). The data shown depict the dynamics of primary- and recall-lymphocyte infiltration responses in group B broilers, which were not vaccinated *in ovo*, and group A broilers, which received the same vaccines *in ovo* on embryonic day 18 during phase 1. Leukocyte populations in GF-pulp cell suspensions were identified by immunofluorescent staining with fluorescence-conjugated, chicken leukocyte-specific mouse monoclonal antibodies, followed by fluorescence-based flow cytometry. Leukocyte data were analyzed using two-way ANOVA (treatment (trt), time, and their interaction (IxN)) and are presented as mean percentage of total pulp cells (% pulp cells) ± SEM. When IxN were significant (*p* ≤ 0.05), W–Z: for each trt, timepoints that do not share the same letter are different, and a–c: trt that do not share the same letter within a timepoint are different. When IxN were not significant (*p* > 0.05), W–Z or a, b: main effects of timepoint or trt that do not share the same letter are different.

For infiltration of B lymphocytes into GF-pulp tissues following i.d. injection of vaccines, there were trt × time interactions (*p* < 0.0001) for both the primary and recall responses (Figure 6). In sham-vaccinated broilers, B cell proportions (% total pulp cells) did not change over time (*p* < 0.05) during either response. B cell proportions in both eB and FK groups increased (*p* < 0.05) to maximal levels at 24 h PV and remained elevated through 3 d PV or 5 d PV in the primary or secondary response, respectively, before returning to pre-injection (0 h) levels at 7 d PV. During the primary response, B cell proportions in B-eB and B-FK were higher (*p* < 0.05) than in B-Sham from 24 h to 3 d PV, and B cell levels in B-FK were greater (*p* < 0.05) than in B-eB at 24 h and 48 h PV, but not different (*p* > 0.05) from each other at 3 d to 7 d PV. During the recall response, B cell levels in the A-eB group were greater than in the A-Sham group from 24 h through 5 d PV, and B cell levels in the A-FK group were greater than in A-eB at 24 h, not different from A-eB at 48 h, lower than A-eB at 3 d, and different from A-eB at 5 d PV.

Distinct patterns in the dynamics of the primary and recall responses were observed regarding the proportions of total T cells (Figure 6). The primary T cell response to intradermal vaccination in GF pulps showed significant main effects of treatment (*p* < 0.05) and time (*p* < 0.0001), but no trt × time interactions. Overall, T cell proportions increased (*p* < 0.05) to peak levels at 24 h PV, remained below but near this level at 48 h and 3 d, then decreased, reaching pre-injection levels at 7 d PV. Throughout this time course, B-FK had higher (*p* < 0.05) total T cell percentages than B-Sham, while levels in the B-eB group were between those of B-FK and B-Sham and not different from either group. For the recall response, there were trt × time interactions (*p* < 0.05). Total T cells in the A-Sham group did not change over time (*p* > 0.05) PV. Total T cell levels increased (*p* < 0.05) in both A-eB and A-FK groups to maximal levels at 24 h PV; thereafter, T cells in the A-eB group remained at this level through 5 d and those in the A-FK group gradually decreased (*p* < 0.05) to levels still above pre-injection on 5 d and returning to pre-injection levels at 7 d PV (*p* > 0.05). The proportions of total T cells in GF pulps were similar in A-eB and A-FK throughout, except at 3 d when the A-FK group’s levels were between those of A-eB and the A-Sham group, and not statistically different from each other.

There were no trt by time interactions for the infiltration response of CD4^+^ T cells to the i.d. vaccinations (Figure 6), and both primary and recall responses displayed a similar trend with main effects of time (*p* < 0.0001) and treatment (Group B, *p* < 0.01; Group A, *p* < 0.0001). Independent of treatment, the proportion of CD4^+^ T cells had increased (*p* < 0.05) to maximal levels by 24 h PV and, in the primary response, remained at this level through 3 d PV before gradually decreasing (*p* < 0.05) to pre-injection levels by 7 d PV; whereas in the recall response, CD4^+^ T cell levels persisted until 5 d PV before dropping (*p* < 0.05) to pre-injection levels at 7 d PV. The proportions of CD4^+^ T cells were greater (*p* < 0.05) in the eB and FK groups than in the sham group at 24 h to 3 d and 24 h to 5 d for the primary and recall responses, respectively, while CD4+ T cell levels in the eB and FK groups did not differ (*p* > 0.05) throughout the examination periods. statistically.

For CD8^+^ T cells, there were trt × time interactions (*p* < 0.0001) for both the primary and recall responses to the i.d. vaccine administration (Figure 6). In both cases, CD8^+^ T cell proportions in the sham groups did not change over time. In the primary response, CD8^+^ T cell proportions increased to maximal levels at 24 h PV in both the B-eB and B-FK groups. However, in the B-eB group, CD8^+^ T cells returned to their lowest levels by 7 d PV and were only significantly greater at 24 h compared to 7 d PV; whereas in the B-FK group, CD8^+^ T cell levels dropped at 48 h to levels still above 0 h, and remained numerically above pre-injection levels thereafter. At all timepoints, the CD8^+^ T cell proportions of the B-eB and B-FK treatments were similar to those of B-Sham, except at 24 h, where B-FK had higher (*p* < 0.05) proportions of CD8^+^ T cells than both B-eB and B-Sham, which were statistically similar. In the recall response, the CD8^+^ T cells reached maximal levels at 24 h PV in both the A-eB and A-FK groups, whereby levels in the A-eB group remained at (48 h) or near (3 d and 5 d) this level, while levels in the A-FK group dropped to lower levels at 48 h and remained at this level until 5 d PV. In both the A-eB and A-FK groups, the proportions of CD8^+^ T cells in the GF pulps returned to pre-injection levels at 7 d PV. The CD8^+^ T cell levels in the A-eB and A-FK groups were greater (*p* < 0.05) than those in the A-Sham group from 24 h to 5 d and 24 h to 3 d PV, respectively.

There were no trt by time interactions for the γδ T cell response to the i.d. vaccinations (Figure 6), but there were main effects of time (*p* < 0.0001) for both primary and recall responses, and a main effect of treatment (*p* < 0.001) for the recall response. Independent of treatment, γδ T cells gradually increased to peak levels at 48 h PV, then dropped to pre-injection levels at 3 d to 7 d PV. During the recall response, γδ T cells increased (*p* < 0.05) at 24 h, reached peak levels at 48 h PV, then dropped slightly (*p* < 0.05) but remained elevated at 3d and returned to 24 h levels at 5 d before dropping to pre-injection levels at 7 d. Throughout, the A-eB and A-FK vaccination groups had greater (*p* < 0.05) γδ T cell levels than the A-Sham group and were not different (*p* > 0.05) from each other. Side-by-side comparison of the magnitude of the γδ T cell primary and recall responses suggests higher levels of γδ T cells in the recall response. In summary, B-eB had elevated proportions of heterophils and macrophages in GF pulps by 6 h PV, compared to the other groups in the primary response. The dynamics of total leukocytes, total lymphocytes, B cells, total T cells, and CD4^+^ T cells were similar, with both eB and FK groups showing elevated proportions compared with sham in both primary and recall responses. Recall responses had higher cell proportions at the corresponding timepoints compared to the primary responses, indicating immune memory.

### 3.5. Changes in Leukocyte Concentrations in the Blood of Broiler Chickens in Response to Primary or Secondary Intradermal Vaccine Administration into Growing Feather Pulps

**Figure 7:**
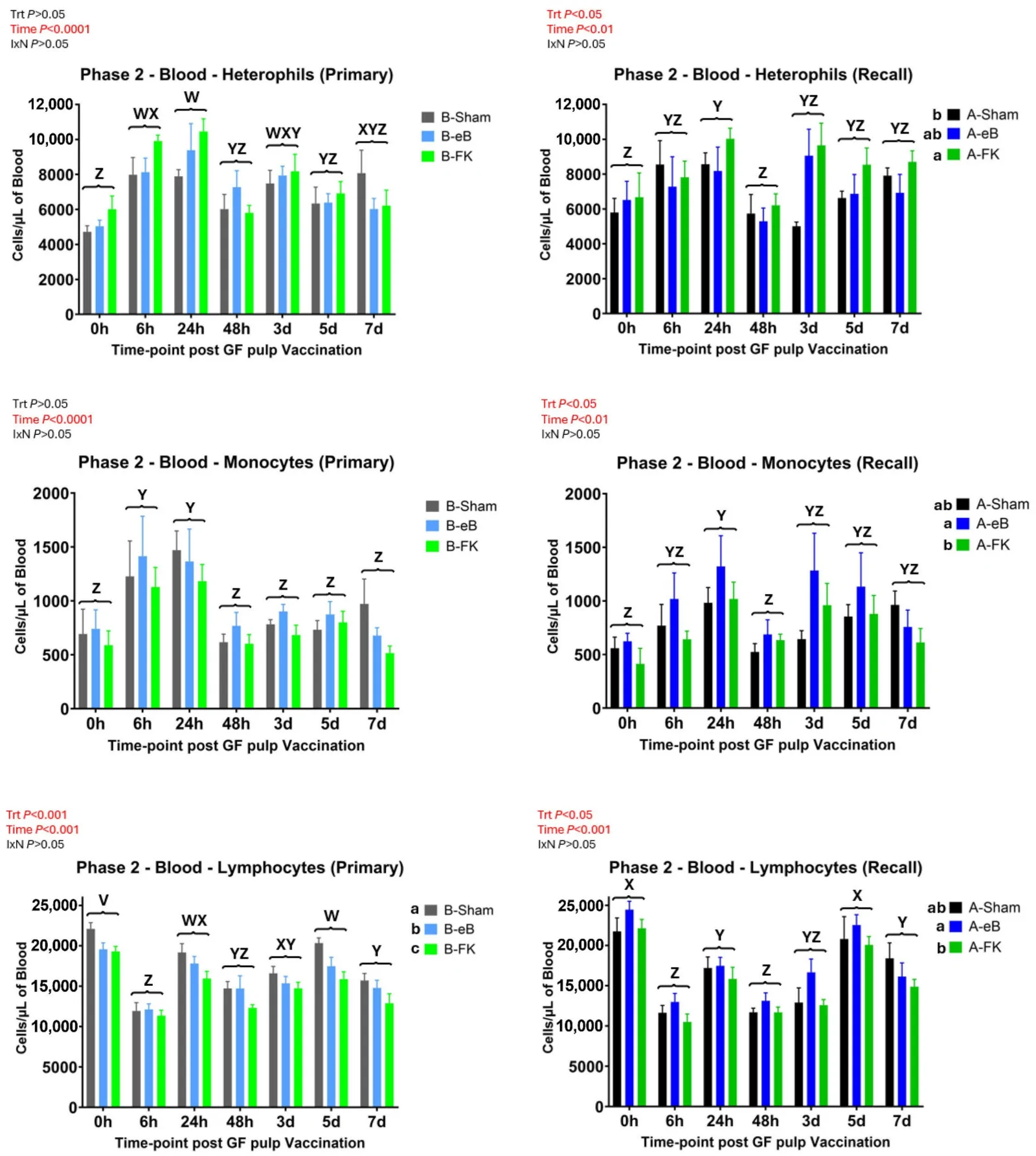
Concentrations of heterophils, monocytes, and total lymphocytes in the blood of 5-week-old chickens before (0 h) and at 6 h, 24 h, 48 h, 3 d, 5 d, and 7 d after intradermal vaccination (PV) with either eBeam-killed *Staphylococcus aureus* (A-eB, B-eB), formalin-killed SA (A-FK, B-FK), or vehicle (A-Sham, B-Sham) vaccines. The vaccines were administered by intradermal (i.d.) injection of growing feather (GF) pulps (10 μL/GF, 22 GF/bird). The data shown depict the dynamics of primary- and recall-blood leukocyte responses in group B broilers, which were not vaccinated *in ovo*, and group A broilers, which received the same vaccines *in ovo* on embryonic day 18 during phase 1. Up to 1.5 mL of heparinized blood was collected from the same 5 birds per treatment at each timepoint. Leukocyte populations in whole blood were identified by immunofluorescent staining with fluorescence-conjugated, chicken leukocyte-specific mouse monoclonal antibodies, followed by fluorescence-based flow cytometry. Data were analyzed using repeated measures two-way ANOVA (treatment (trt), time, and their interaction (IxN)) and are presented as mean leukocyte concentrations (cells/µL of blood) ± SEM. W–Z: main effects of timepoints that do not share the same letter are statistically different, and a–c: main effects of trts that do not share the same letter are statistically different.

For the blood heterophil concentrations (Figure 7) there were main effects of time (*p* < 0.0001) in group B birds during the primary response to eB, FK, and sham intradermal vaccines. In contrast, for the recall response in group A broilers, there were significant main effects of both time (*p* < 0.01) and treatment (*p* < 0.05). No trt × time interaction effects were observed in either case. Independent of treatment, heterophil concentrations in group B broilers (primary response) increased from 0 d to the highest levels at 24 h, then fluctuated (*p* < 0.05), with concentrations dropping to near pre-injection levels at 48 h and 5 d PV, and increasing again to near 6 h levels on 3 d and 7 d PV. The heterophil concentrations at 6 h, 24 h, and 3 d PV were higher (*p* < 0.05) than before i.d. vaccination (0 h). Independent of treatment, heterophil concentrations in group A (recall response) broilers increased (*p* < 0.05) from 0 h to 24 h PV. At 48 h PV, the heterophil concentrations dropped to pre-injection levels and increased again to levels intermediate to those before injection (0 h) and the maximal levels at 24 h PV from 3 d to 7 d. Overall, the A-FK group had higher (*p* < 0.05) heterophil concentrations throughout the time course than the A-Sham group, with heterophil concentrations of the A-eB group between those of A-FK and A-Sham, but not significantly different from either.

There were no trt × time interaction effects on the blood monocyte concentrations (Figure 7) for the primary and recall vaccination responses. However, there were significant (*p* < 0.0001) main effects of time in the primary response of group B birds, and main effects of time (*p* < 0.01) and treatment (*p* < 0.05) in the recall response for group A broilers. During the primary response, all treatment groups had higher (*p* < 0.05) monocyte concentrations at 6 h and 24 h PV than before injection (0 h) and from 48 h to 7 d PV. Throughout the recall response, the A-eB group exhibited significantly higher monocyte concentrations than the A-FK group, while levels in the A-Sham group were between those of the A-eB and A-FK groups, but not different from either. Considering the main effects of time, monocyte concentrations increased (*p* < 0.05) from 0 h to 24 h, then dropped (*p* > 0.05) to pre-injection levels at 48 h PV, and increased (*p* > 0.05) again at 3 d PV to levels between 0 h and 24 h PV, where they remained until 7 d PV.

For both the primary (group B) and recall responses (group A) there were no trt × time interaction effects on the total lymphocyte concentrations (Figure 7). However, both primary and recall responses showed significant main effects of time (*p* < 0.001) and treatment (*p* < 0.001 for group B; *p* < 0.05 for group A). Both primary and recall responses showed alternating concentrations of total lymphocytes, starting with maximum concentrations at 0 h, dropping to minimum concentrations at 6 h, increasing to near but below pre-injection levels (0 h) at 24 h, dropping to 6 h levels at 48 h, then increasing to (A-group) or just below (B-group) pre-injection levels by 5 d, and dropping again on 7 d PV to levels that were between and different from both the highest and lowest lymphocyte concentrations. During the primary response, B-Sham broilers had the highest total lymphocyte concentrations, followed by B-eB, which was significantly lower than B-Sham; B-FK had the lowest (*p* < 0.001) total lymphocyte concentration compared with the other two groups. Throughout the recall response, A-eB had higher (*p* < 0.05) total lymphocyte concentrations than A-FK. A-Sham had intermediate lymphocyte concentrations between A-FK and A-eB, but the differences were not significant.

**Figure 8:**
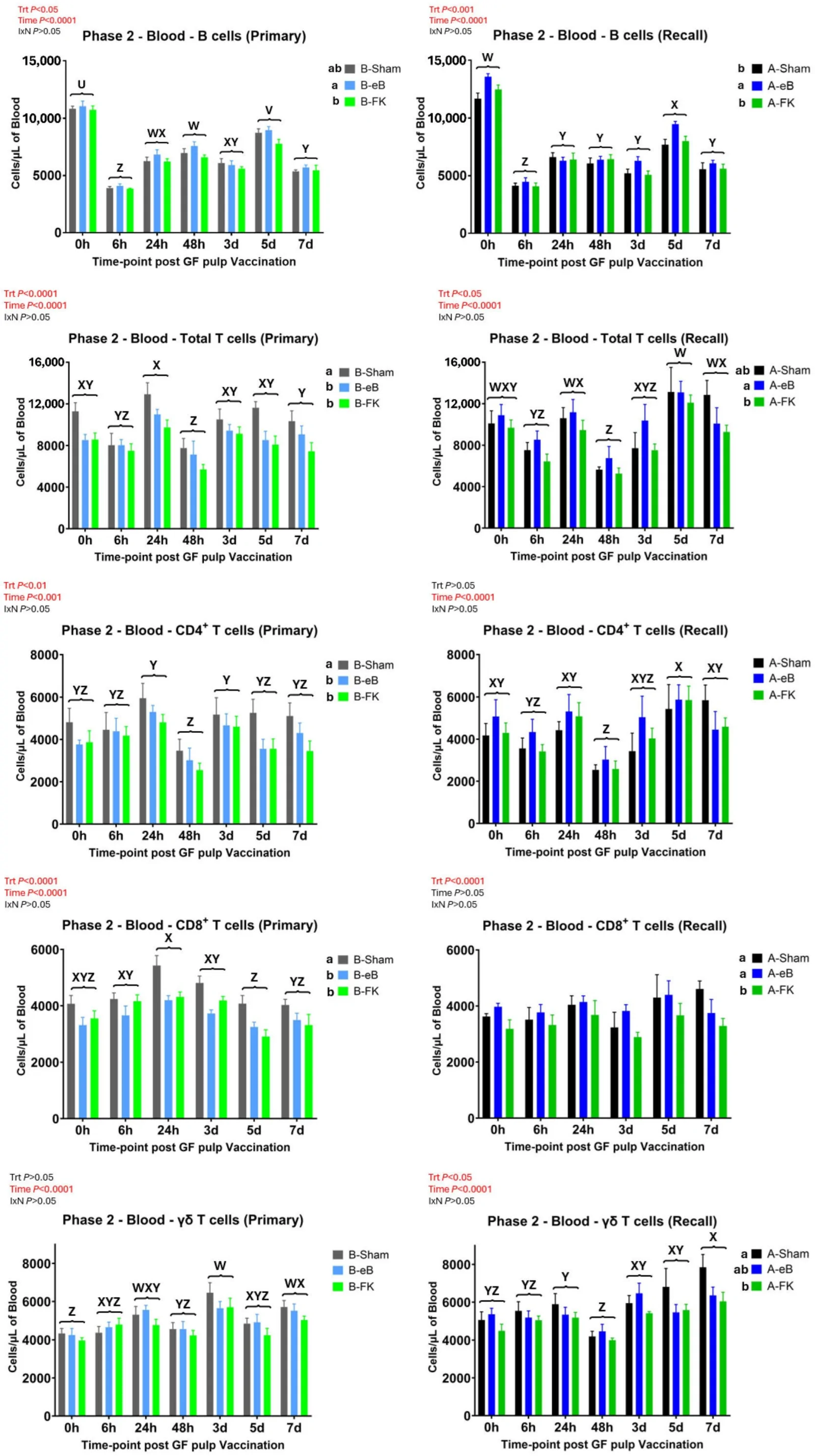
Concentrations of various lymphocyte populations (B cells, T cells, CD4^+^ T cells, CD8^+^ T cells, and γδ T cells) in the blood of 5-week-old chickens before (0 h) and at 6 h, 24 h, 48 h, 3 d, 5 d, and 7 d after intradermal vaccination (PV) with either eBeam-killed *Staphylococcus aureus* (A-eB, B-eB), formalin-killed SA (A-FK, B-FK), or vehicle (A-Sham, B-Sham) vaccines. The vaccines were administered by intradermal (i.d.) injection of growing feather (GF) pulps (10 μL/GF; 22 GF/bird). The data shown depict the dynamics of primary- and recall-blood leukocyte responses in group B broilers, which were not vaccinated *in ovo*, and group A broilers, which received the same vaccines *in ovo* on embryonic day 18 during phase 1. Up to 1.5 mL of heparinized blood was collected from the same 5 birds per treatment at each timepoint. Leukocyte populations in whole blood were identified by immunofluorescent staining with fluorescence-conjugated, chicken leukocyte-specific mouse monoclonal antibodies, followed by fluorescence-based flow cytometry. Data were analyzed using repeated measures two-way ANOVA (treatment (trt), time, and their interaction (IxN)) and are presented as mean leukocyte concentrations (cells/µL of blood) ± SEM. U–Z: main effect means of timepoints that do not share the same letter are statistically different, and a, b: main effects of trts that do not share the same letter are statistically different.

Analysis of the concentrations of various lymphocyte populations revealed no trt × time interactions (*p* > 0.05) for any of the lymphocyte populations examined (B cells, total T cells, CD4^+^ T cells, CD8^+^ T cells and γδ T cells) in both the primary (group B) and recall (group A) responses (Figure 8). For B cells, there were main effects of time (*p* < 0.0001) for both primary and recall responses. In both group B and A broilers, B cell concentrations dropped substantially (*p* < 0.05) to the lowest levels at 6 h PV, then increased (*p* < 0.05) at 24 h and remained near (B group) or at (A group) this level through 3 d PV, then increased (*p* < 0.05) further at 5 d to levels still below 0 h and dropped (*p* < 0.05) again to 3 d concentrations at 7 d PV. The B cell concentrations at 24 h, 48 h, 3 d, and 7 d PV were higher than at 6 h, but lower than at 5 d PV. There were also main effects of treatment (primary, *p* < 0.05) and recall (*p* < 0.001) on the B cell concentrations. Throughout the primary response, broilers in the B-eB group had higher B cell concentrations than the B-FK group, while levels in the B-Sham group were between those of the B-FK and the B-eB groups but not different (*p* > 0.05) from either group. During the recall response, B cell concentrations in A-eB broilers were higher (*p* < 0.05) compared to those in both the B-FK and B-Sham broilers, which had similar (*p* < 0.05) overall B cell concentrations.

The total T cell concentrations in the blood showed alternating cell concentrations across timepoints (time main effect *p* < 0.0001) for both the primary and recall vaccine responses (Figure 8). Independent of treatment and type of response, total T cell concentrations dropped numerically from 0 h to 6 h, then increased (*p* < 0.05) above 6 h levels at 24 h, and dropped (*p* < 0.05) to the lowest levels at 48 h. Thereafter, T cell concentrations returned (*p* < 0.05) to pre-injection levels by 3 d and remained at these levels through 7 d, for both the primary and recall responses. Overall, in the primary response (trt main effect *p* < 0.0001), the total T cell concentrations were greater in B-Sham broilers than in the B-eB and B-FK broilers, which were not different from each other, whereas in the recall response (trt main effect *p* < 0.0001), the total T cell concentrations were higher with A-eB than A-FK, and neither was different from A-Sham.

The time course of the CD4^+^ T cell subsets followed a pattern similar to that of total T cells, with significant main effects of time for both responses (*p* < 0.001). Independent of treatment and type of response, their concentrations fluctuated around pre-injection levels at 6 and 24 h PV, dropped (*p* < 0.05) to the lowest levels at 48 h, then returned (*p* < 0.05) to pre-injection levels at 3 d and remained at this level through 7 d (Figure 8). The main effects of treatment were significant for the primary response (*p* < 0.01), but not for the recall response (*p* > 0.05), with higher CD4^+^ T cell concentrations in the B-Sham group than in both the B-eB and B-FK groups, which were similar to each other.

Regarding the systemic CD8^+^ T cell concentrations (Figure 8), both primary and recall responses showed significant main effects of treatment (*p* < 0.0001). For the primary response, there was also a main effect of time (*p* < 0.0001), with CD8^+^ T cell concentrations gradually increasing from 0 h to the highest levels at 24 h, followed by a decline. The concentrations of CD8^+^ T cells at 6 h, 24 h, and 48 h PV were higher than at 5 d PV. CD8+ T cell concentrations did not change over time (*p* > 0.05) during the recall response. During the primary response, B-Sham had higher (*p* < 0.05) CD8^+^ T cell concentrations than B-eB and B-FK broilers, which had similar (*p* < 0.05) CD8^+^ T cell concentrations. During the recall response, both A-eB and A-Sham groups had similar CD8^+^ T cell concentrations that were greater (*p* < 0.0001) than those in the A-FK.

There were significant main effects of time (*p* < 0.0001) on the concentrations of γδ T cells (Figure 8) for both the primary and recall responses (Figure 8). During both responses, γδ T cell concentrations increased (*p* < 0.05) from 0 h to 24 h and returned to near pre-injection levels at 48 h. By 3 d PV, γδ T cells reached the highest (*p* < 0.05) levels in the primary response, then dropped slightly at 5 d and returned to near peak concentration at 7 d PV. In the recall response, γδ T cell concentrations also increased (*p* < 0.05) again by 3 d PV but continued to increase to the highest (*p* < 0.05) levels by 7 d. The main effects of treatment (*p* < 0.05) were significant for the recall response, but not for the primary response. During the recall response, A-Sham broilers had higher (*p* < 0.05) γδ T cell concentrations than the A-FK broilers, while levels in A-eB broilers were between those of A-Sham and A-FK and not different from either group. In summary, A-FK had a significantly higher heterophil response, while A-eB had significantly higher macrophage-, total lymphocyte-, and total T cell responses during the recall immune reaction in blood than the other groups. The eB groups had the significantly highest B cell concentrations in blood during both primary and recall responses. Primary leukocyte responses in blood were similar across all treatments for heterophils and monocytes, but the sham group showed elevated T cell responses.

### 3.6. Staphylococcus Aureus (SA)-Specific IgM, IgY, and IgA Antibody Levels in the Plasma of 35-Day-Old Broilers After Primary or Secondary Intradermal Injections of SA-Sham, -eB, or -FK Vaccines into Growing Feather Pulps

**Figure 9:**
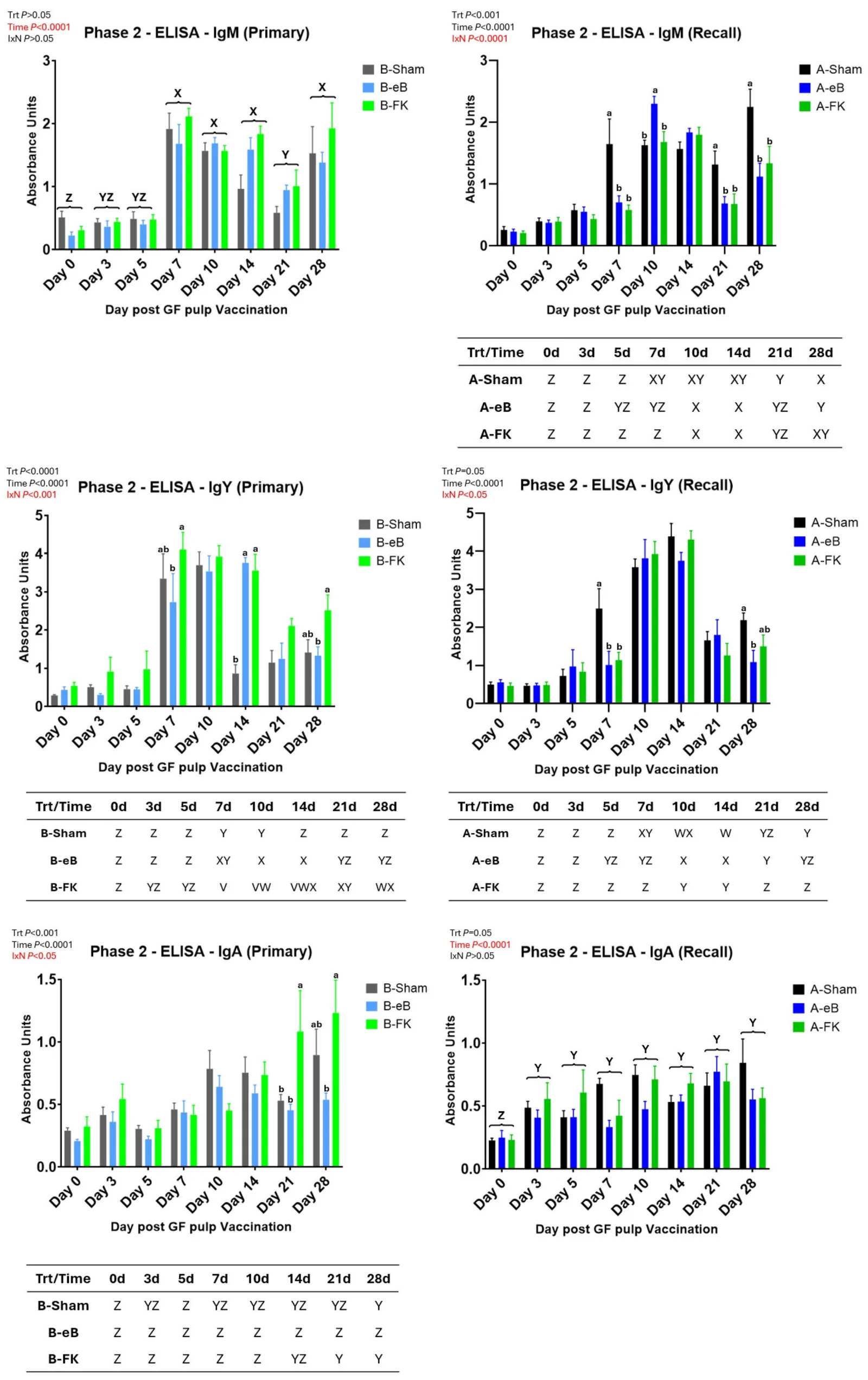
*Staphylococcus aureus* (SA)-specific IgM, IgY, and IgA levels in the blood of 5-week-old group A and B broiler chickens in response to intradermal vaccination with eBeam-killed SA (A-eB, B-eB), formalin-killed SA (A-FK, B-FK), or vehicle (A-Sham, B-Sham) vaccines before (0 d) and on days 3, 5, 7, 10, 14, 21, and 28 post-vaccination (PV). The vaccines were administered by intradermal (i.d.) injection of growing feather (GF) pulps (10 μL/GF; 22 GF/bird). The data shown depict the dynamics of primary- and recall-antibody responses in group B broilers, which were not vaccinated *in ovo*, and group A broilers, which received the same vaccines *in ovo* on embryonic day 18 during phase 1. Up to 1.5 mL of heparinized blood was collected at each timepoint from the same 5 birds per treatment. ELISA was used to quantify the relative levels of SA-specific antibodies. Two-way repeated-measures ANOVA was used to analyze the effects of treatment (trt), time, and their interaction (IxN), and the results are presented as mean absorbance units ± SEM. When interactions were significant (*p* ≤ 0.05), W–Z: timepoints are significantly different within the considered trt unless they share the same letter, and a, b: trts are significantly different within the considered timepoint unless they share the same letter. When interactions were not significant (*p* > 0.05), X–Z: statistical differences between the main effects of timepoints are denoted by different letters.

Relative SA-specific antibody levels (a.u.) in the blood were evaluated for birds of both groups A and B in response to intradermal (i.d.) GF-pulp injections of either sham, SA-eB, or -FK vaccines at 35 days of age. For each group, the specific IgM, IgY, and IgA levels in blood were evaluated via ELISA before (day 0) and on days 3, 5, 7, 10, 14, 21, and 28 PV. Thus, the antibody responses to the vaccines were a primary immune response in birds in group B that had not been exposed to the vaccine before, but a secondary (recall) immune response in broilers in group A that had already received the same vaccines *in ovo*. Figure 9 below depicts trends in the changes in SA-specific antibody levels in response to intradermal injections into the GF pulps.

For SA-specific IgM levels in group B (primary response) there was a significant main effect of time (*p* < 0.0001), while the main effects of treatment and the treatment × time interaction were not significant. Independent of treatment, IgM levels remained near pre-injection levels before increasing to maximal levels on 7 d, remaining at this level on 10 d and 14 d, before dropping (*p* < 0.05) on 21 d to above baseline levels and rebounding to maximal levels on 28 d. For the recall response, the trt × time interaction for IgM levels were significant (*p* < 0.0001). Similar to the primary response, pre-injection levels were sustained until 5 d PV in all treatments. For the A-Sham group, circulating IgM levels was highest (*p* < 0.05) on 7 d, 10 d, 14 d, and 28 d PV, while the levels on 21 d were lower than on 28 d but significantly higher than pre-injection levels. Treatment differences were observed on 7 d, 10 d, 21 d, and 28 d PV, whereby levels in A-eB and A-FK broilers were lower (*p* < 0.05) than in A-Sham broilers on 7 d, 21 d, and 28 d PV, and on 10 d, levels were higher in A-eB than in A-Sham and A-FK broilers.

For SA-specific IgY levels in the blood during both the primary (group B) and recall (group A) responses, there were treatment-by-time interaction effects (*p* < 0.001 and *p* < 0.05, respectively). Independent of treatment, both primary and recall responses showed a general trend of maintaining pre-injection IgY levels from 0 d through 5 d PV, then increasing (*p* < 0.05) to maximal levels on 7 d, remaining at this level through 14 d, before dropping to lower than maximal levels but still above pre-injection levels on 21 d and 28 d. In the primary response, B-FK had significantly higher IgY levels on 7 d and 28 d compared to the other treatments. On these 2 days, IgY levels in the B-Sham group were intermediate to and not different from those of B-FK and B-eB broilers. However, on 14 d, IgY levels in both B-eB and B-FK were higher (*p* < 0.05) than in B-Sham broilers, but not different from each other. During the recall response, IgY levels in A-Sham broilers were higher (*p* < 0.05) than in the other 2 groups on 7 d. On 28 d, A-Sham IgY levels were higher than in A-eB broilers, and levels in A-FK broilers were between those in A-Sham and A-eB broilers but not different from either group (*p* > 0.05).

For plasma SA-specific IgA levels, there was a trt × time interaction effect (*p* < 0.05) for the primary response. The B-Sham group showed a gradual increase in circulating IgA over time, reaching higher levels (*p* < 0.05) than pre-injection levels on 28 d. The B-eB group also showed a gradual numerical (*p* > 0.05) increase in IgA levels over the 28-day sampling period, while in B-FK broilers, plasma IgA increased to maximal levels on D21 and D28 that were higher (*p* < 0.05) than on 0 d through 10 d. On 21 d, the B-FK group had higher (*p* < 0.05) IgA levels than the other 2 groups, which did not differ from each other. On 28 d, IgA levels in B-FK were higher than in B-eB broilers, and levels in B-Sham broilers were between those of the other two vaccine treatments but were not different (*p* < 0.05) from either. For the recall response, there were only main effects of time (*p* < 0.0001); independent of treatment, plasma SA-specific IgA levels were already higher (*p* < 0.05) on 3 d than 0 d, and continued to gradually, but numerically (*p* > 0.05), increase throughout the 28-day sampling period. In summary, IgM and IgY levels of all groups increased early in the time course, by either 7 d or 10 d, and decreased by 21 d PV, while IgA increased gradually from 0 d through 28 d in both primary and recall responses.

## 4. Discussion

Avian immunity in response to *Staphylococcus aureus* (SA) infections [44,45,46] as well as selected SA antigens [47,48] has been investigated under different experimental settings. This includes determining SA inoculation route-related changes in immune responses in layers [46], assessing the effects of starvation on avian immune responses [44], and evaluating the suitability of using exposure to SA to prime immunity in chickens against other pathogens and diseases [45,47,48]. However, limited knowledge exists on the quantitative and temporal characteristics of systemic (blood) humoral and cellular, as well as local cellular (GF pulp) immune responses against SA in broiler chickens, a major pathogen that adversely impacts poultry welfare [49,50,51], industry revenue, and consumer safety via foodborne infections [1,52]. In poultry, using the patented GF-pulp cutaneous bioassay [31], together with blood analyses, provides a clear visualization of local responses in living tissues (GF pulps) and systemic immune responses to the i.d. vaccination reflected in the blood. This dual-window approach provides insights that could inform the development of prospective preventive measures for SA infections in chickens.

### 4.1. Anti-Staphylococcus Aureus Immune Responses of In Ovo Vaccinated Broilers

The SA-specific antibody responses to *in ovo* and i.d. vaccinations resulted in distinct IgM, IgY, and IgA antibody presence. In newly hatched chicks vaccinated *in ovo*, regardless of treatment, IgY levels were already elevated at D3 PV (day of hatch) and remained elevated until D11 PV, then declined at D18 PV. This high level of SA-specific IgY in all treatments already at D3 PV might indicate passive immunity conferred by maternal antibodies, which may be masking the active SA-specific IgY response in the vaccinated chicks. Hamal et al. (2006) [53] established a direct relationship between maternal antibody levels in the offspring’s circulation and those in the hens’ plasma or eggs, which is important during the chick’s first few weeks of life, as their immune system is underdeveloped. Following *in ovo* vaccinations, SA-specific IgM levels across all treatments remained low from D3 PV onwards, then increased to their highest on D31 PV; however, on D7 PV, A-eB was higher than A-Sham and A-FK. In chicks during the first week after hatch, circulating IgM is low, and it is estimated that less than 1% is maternally transferred [53]. While their immune system is developing and relies on passive immune protection via maternal IgY, IgM, a pentamer, is the first to be self-synthesized, providing early systemic active defense against invaders [54,55]. Hence, elevated SA-specific IgM levels on D7 PV in the A-eB group suggest stronger humoral immune activation, in which IgM could neutralize SA via opsonization and activate the complement system to assist in pathogen clearance early in life. For SA-specific IgA between D3 and D31 PV, peak antibody levels were recorded on D7 PV in the present study for the A-eB and A-FK groups. On D5 and D7 PV, A-eB had higher IgA levels than the other treatments. The mucosal immune system in poultry serves as the first line of defense against airborne infections, with secretory IgA being the primary antibody [56]. Maternal IgA, although present in the eggs at approximately 1 mg, is not taken up into the chick’s circulation [53]. Accordingly, early (D7 PV or D4 post-hatch), significantly higher SA-specific IgA levels elicited by eBeam-killed SA may reflect superior protection at mucosal surfaces early in the life of the chicks, when administered *in ovo*. Assumpcao et al. (2024) [18] also reported the ability of eBeam- and formalin-killed multi-strain *Staphylococcus* vaccines to elicit high specific serum-IgA and IgM profiles in broiler chickens. While SA can spread via aerosol, particularly within the intensive, crowded environments of poultry houses, aerosolized bacteria often result in respiratory tract-mediated acquisition by chickens, eventually leading to infection [57,58]. Skin wounds, minor surgical procedures (trimming of the beak, toe, or comb), vaccine injections, contamination of an open navel, and compromised intestinal mucosa can introduce *Staphylococcus* into local tissues or the bloodstream of juvenile chicks [59]. Therefore, the early IgM- and IgA-mediated protection against *Staphylococcus* provided by eBeam-killed SA *in ovo* vaccination may help protect chicks from infections associated with inevitable environmental stressors during early life, leading to decreased mortality, reduced pathogenic stress, and improved performance.

Heterophils (the avian counterpart to mammalian neutrophils) are the initial line of defense and the first phagocytes to arrive at inflamed tissues; however, they have a short lifespan [42]. Monocytes/macrophages generally arrive more gradually than heterophils but are long-lived and display a sustained presence and a variety of functional responses. Because of an extensive array of pattern recognition receptors, macrophages have a greater ability to respond to a variety of microbes by producing inflammatory mediators, killing phagocytosed intracellular microbes, initiating tissue healing, and restoring tissue homeostasis [60,61]. As a result, heterophil levels increase rapidly in the blood and are closely followed by an increase in monocytes, which subsequently migrate into tissues to act as macrophages [62,63]. This trend is reflected in the heterophil and monocyte responses in the blood of newly hatched chicks following *in ovo* vaccination (Figure 3). All groups showed a drop in blood heterophil concentrations at D7 PV compared to D3, D5, and D11 PV, while monocyte concentrations were lowest on D5 PV (D2 of hatch), followed by an elevation on D7 PV when heterophils declined. As A-Sham followed the same trend as A-eB and A-FK in heterophil and monocyte concentrations over the 11-day PV period, these dynamics may be attributable to development-related proliferation of phagocytes during the first week of hatch. Typically, to cope with the rapid adjustment to their new environment, phagocytic activity is high in newly hatched chicks. This primarily involves heterophils, followed gradually by monocyte activity, showing extensive differentiation and proliferation of innate immune cells during the first few days post-hatch [64] while maintaining higher heterophil/lymphocyte ratios [65,66] until lymphocytes mature enough to drive adaptive immunity. Therefore, the trend in blood heterophil and monocyte responses in the present study following *in ovo* vaccinations in newly hatched chicks may reflect the combined effects of development and a response to the SA immunizations.

Independent of treatment, the dynamics of blood B cells and T cells (total T cells, CD4^+^ T cells, CD8^+^ T cells, and γδ T cells) in response to the *in ovo* SA vaccines (Figure 4) generally showed lower concentrations in the circulation at D3 and D5 PV (day of hatch and 2 days later) compared to D7 and D11 PV when their concentrations increased significantly. The lower mononuclear cell concentrations observed until 48 h post-hatch may be explained by the rapid water intake by newly hatched chicks, which directly counteracts dehydration-induced high blood viscosity (hemoconcentration) by expanding plasma volume, resulting in a more balanced, lower hematocrit, and improved overall health [67,68]. In contrast to T cells and their sub-categories, which depict only main effects of time across all treatments, the circulating B cells show interaction effects of treatment and time, where the B cell levels across all treatments are similar until D7, but differ on D11, with A-eB and A-FK groups showing significantly higher concentrations compared to A-Sham. This closely mimics total blood lymphocyte concentrations, with significantly higher concentrations in the A-eB and A-FK groups than in the A-Sham group by D11. This observation, coupled with the significant increase in IgM and IgA profiles on D7 in the A-eB group, may reflect a B lymphocyte-mediated response against *in ovo*-injected killed SA, initiated by the eBeam vaccine. This is also visible in a previous study [18] where the IgA levels of eBeam- and formalin-killed *Staphylococcus*-vaccinated broilers were elevated on D33 PV compared to D11 PV, which was also reflected by the increased blood-B cell concentrations of the vaccinated birds on D33 over D11 PV.

### 4.2. Local Leukocyte Profiles in Response to Primary and Recall Intradermal Staphylococcus Aureus Injections into GF Pulps

Intradermal injections of killed SA into growing feather (GF) pulps, a skin derivative of birds, were carried out during phase 2 (P2) of the present study to gain insights into the tissue/cellular responses occurring locally, or at the site of injection [31]. In P2, after i.d. vaccinations into the GF pulps, both local heterophil and macrophage proportions within GF pulps increased at 6 h PV (Figure 5). While heterophil levels (% of pulp cells) rose at 6 h and then rapidly declined, macrophage levels alternated between high and low but remained elevated above baseline even at 5 d PV. Santamaria et al. (2025) [32] describe the influx of heterophils and macrophages in response to PGN injections into GF pulps, with maximal levels observed at 6 h and a return to baseline by 72 h and 24 h, respectively. Similarly, heterophils also dominated the infiltration response, reaching peak levels at 6 h PV followed by a steep decline in GF pulps of chickens i.d. injected with *Mycobacterium butyricum* [69]. In contrast, macrophages within GF pulps gradually increased to maximal levels by 3 d PV and then gradually declined but remained elevated even at 7 d PV. Therefore, the present study’s heterophil and macrophage response to i.d. GF pulp vaccinations show classic infiltration dynamics of an innate inflammatory response to sterile injection and microbial infection. While both eB and FK show higher proportions of macrophages than sham at 5 d PV, eB shows higher infiltration of macrophages and heterophils at 6 h PV than both other groups during the primary response. This may indicate a stronger local inflammatory response elicited by the eBeam-killed SA bacteria during the initial pathogen encounter, owing to better retention of immunogenic epitopes. This early inflammatory response in chickens initiated by an *in ovo* eBeam SA vaccine may explain improved survivability, as heterophils and macrophages may confer resistance until adaptive immunity is fully developed, enabling selective elimination of the pathogens [65].

Interestingly, both primary and recall responses in GF pulps during P2 are dominated by lymphocytes, reaching higher levels than those of heterophils and monocytes. Specifically, total lymphocytes in GF pulps (Figure 5) of i.d. injected broilers (eB and FK groups) were elevated at 24 and 48 h and remained above baseline at 72 h PV. This lymphocyte infiltration pattern closely follows observations in previous studies evaluating local and systemic immune responses to i.d. GF pulp injections of peptidoglycan (PGN) in chickens [32,42]. Moreover, Erf and Ramachandran (2016) [69] illustrate a similar observation in chickens i.d. injected with *M. butyricum* (a Gram-positive bacterium), where lymphocyte proportions in GF pulps remain steadily elevated compared to heterophils and macrophages. Considering that *Staphylococcus aureus* is a Gram-positive bacterium with a thick (20–30 nm) cell wall, primarily composed of a highly cross-linked (∼90%) A3α-type PGN, which accounts for 50–60% of its dry weight [70], the similarity in the heterophil, macrophage, and lymphocyte infiltration pattern observed with SA may be attributed largely to PGN. Hence, PGN of Gram-positive bacteria might play an important role in promoting local lymphocyte infiltration, leading to a rapid adaptive immune response, irrespective of the vaccine formulation. Moreover, in the eB group, the lymphocytes remain steadily elevated in GF pulps from 24 h to 3 d PV and from 24 h through 5 d PV in the primary and recall responses, respectively, as opposed to the alternating high and low lymphocyte levels observed in the FK group, or the baseline levels maintained by the sham group throughout. This observation may also suggest a stronger, more stable immune reaction elicited by the eBeam vaccine.

Examination of the dynamics of individual types of lymphocytes following i.d. injections into GF pulps during P2 showed significantly higher B cell proportions within GF pulps from 24 h to 3 d PV during the primary response, and from 24 h to 5 d PV during the recall response (Figure 6). Similar behaviors of B cell responses were recorded in 11-week-old broilers i.d. injected with SA-derived PGN into GF pulps by Santamaria et al. (2025) [32], where B cells remained at baseline levels at 6 h PV after GF pulp injection with PGN, but increased to significantly greater levels from 24 h through 3 d PV. Furthermore, 12-week-old Light-brown Leghorn (LBL) males immunized with *Mycobacterium butyricum* into the breast muscles, followed by a secondary immunization of either PBS or *M. butyricum* into GF pulps 4 weeks later [69], showed B cell responses in GF pulps similar to those in the present study. Specifically, following secondary vaccination with PBS or *M. butyricum*, B cells in GF pulps did not change from pre-injection levels in the PBS group throughout. Still, they increased significantly from 24 h through 3 d PV in the group injected with *M. butyricum.* This B cell infiltration pattern in response to PGN or Gram-positive bacterins is distinctly different from the B cell responses within GF pulps of both LBL and broiler chickens injected with either killed *Salmonella* vaccines or *Salmonella*-derived lipopolysaccharide (LPS; a major component of outer membranes of Gram-negative bacteria), where B cell levels within GF pulps did not change significantly in response to i.d. LPS injections or to primary or secondary i.d. injections of killed *Salmonella* vaccines [35]. These observations clearly indicate a general preference for a prominent B lymphocyte-mediated response elicited by killed Gram-positive bacteria in chickens, with PGN appearing to be the primary driver. Moreover, in cynomolgus macaques [71], bivalent SA vaccines have been shown to induce cytokine production from naive B cells and antigen-specific memory B cells, in addition to increased antibody responses, demonstrating the prominence of a B cell-mediated response against SA across mammalian models. Determining this in chickens warrants investigation of local cytokine profiles in chickens following i.d. SA vaccines into GF pulps.

T lymphocytes also followed an infiltration pattern similar to total lymphocytes and B cells in both the primary and secondary responses to the i.d. injected SA vaccines (Figure 6). For T cells, the booster effect was also more pronounced and sustained, reaching higher percentages of total T cells within GF-pulp cells than during the primary response. During both primary and recall responses, CD4^+^ T cells exhibited the same dynamics as total T cells. They accounted for the largest proportion of infiltrating T cells, with higher CD4^+^ T cell percentages in GF pulps than in CD8^+^ and γδ T cells. This pattern was also prominent in GF pulps of LBL chickens having received *Mycobacterium butyricum* injections into GF pulps [69]. The CD4^+^ T cell-dominated local response to the killed SA vaccines could also explain the postulated Th cell-mediated isotype switch from IgM to IgY and IgA during P2 of the present study. Interestingly, while at lower levels than CD4^+^ T cells in both SA-initiated responses, the presence of CD8^+^ T cells was more pronounced and prolonged in the recall response, pointing towards the development of a cytotoxic phenotype during memory development. The same dynamics were observed for γδ T cell recruitment, whereby γδ T cell levels were highest at 24 h and 48 h PV in the primary response, independent of treatment [69]. However, during the recall response, γδ T cell levels in the eB and FK groups were higher than in the primary response and sustained from 24 h to 5 d, while the sham group showed the same dynamics and levels as in the primary response. The γδ T cell recruitment pattern in the sham group is typical of an inflammatory response to a sterile injection. In contrast, the response dynamics in the eB and FK groups suggest memory development. In human and murine systems, γδ T cells can become long-lived memory T cells that mount potent recall responses to secondary pathogen infections [72], highlighting a pivotal role in generating antigen-dependent memory responses and protective immunity against bacterial, viral, and parasitic infections [73]. Overall, especially during recall responses, B cell and total T cell levels showed similar quantitative changes over time in the eB and FK groups, supporting specific humoral and cell-mediated memory development to the SA vaccines.

### 4.3. Blood Leukocyte Profiles in Response to Primary and Recall Intradermal Staphylococcus Aureus Injections

Independent of treatment, concentrations of heterophils, monocytes, and total lymphocytes in blood during P2 (Figure 7) showed similar trends in both primary and recall responses. Heterophil concentrations rose above baseline at 6 h and 24 h PV, returning to near (primary response) or to (recall response) pre-injection levels at 48 h, and increasing again to concentrations between peak and baseline levels in both primary and recall responses. The same pattern was observed for monocyte concentrations, except that monocytes remained at pre-injection levels after reaching peak concentrations at 6 h and 24 h during the primary response. The higher levels of these cells in the later stages of the recall response may be driven by cytokines [61] from the adaptive response. While there were no treatment differences in the concentrations of these innate leukocytes during the primary response, in the recall response, the heterophil concentrations were higher with FK than with sham, and eB was not different from either, while for monocytes, eB was greater than FK, and sham was not different from either. These treatment differences in the recall responses may also be driven by inflammatory cytokines produced during the adaptive recall response and indicate the better potential of eBeam- and formalin-killed SA to initiate inflammatory responses.

For all treatments, lymphocyte concentrations decreased markedly at 6 h PV and vacillated between pre-injection and 6 h concentrations in the primary response, not fully returning to pre-injection levels during the 7 d period. Interestingly, in the primary response, the sham group had the highest overall lymphocyte concentrations, followed by eB, and the lowest in FK, with these treatment differences most apparent from 24 h PV onward. A similar time-course profile emerged for the recall response, whereby the lymphocyte concentration fully returned to baseline by 5 d PV, then dropped again above 6 h levels at 7 d PV, independent of treatment. Overall, lymphocyte concentrations were higher with eB than with FK, and sham did not differ from either. A possible explanation for the lower circulating lymphocyte concentrations may be vascular adhesion and/or redistribution of mature lymphocytes to various tissues across the body, in addition to extravasation to the site of vaccination (GF pulps), as explained by Byrne et al. (2024) [42]. This is supported by the observation that intraperitoneal injections of SA or SA-derived PGN in mice increased the expression of endothelial vascular adhesion molecules (integrins) [74]. It is also noteworthy that the trends in blood lymphocyte concentration changes in both primary and recall responses of P2 resemble those of B cells and total T cells, highlighting the major participation of both types of lymphocytes in both the local and systemic anti-*Staphylococcus* immune response. Moreover, the eB groups showed significantly higher B cell concentrations in circulation than their FK counterparts (Figure 8) during both primary and recall responses. When considered cumulatively with the local B cell response in GF pulps (Figure 6), the eBeam vaccine appears to elicit an effective and sustained B cell-mediated immune reaction against SA in chickens. While some vaccine studies in mammals support a B-cell- and antibody-mediated response to *Staphylococcus* infections, SA appears to suppress the quality of the humoral response. In mammals, SA modulates B cell responses through the expression of Staphylococcal Protein A (SpA). This surface protein drives polyclonal B cell expansion and induces their death in the absence of co-stimulation [75,76]. SpA has been shown to bind to IgG on mammalian B cells, causing apoptosis, accelerated preferential deletion of targeted spleen marginal zone B cells, impaired responsiveness to T-independent immunogens [76], and diminished production of antigen-specific, class-switched, affinity-matured antibodies [75] in mice. Interestingly, chicken IgY differs from mammalian IgG in that the Fc domains of IgY show no affinity to several immunoglobulin-binding proteins, including SpA, thereby preventing SpA binding to avian IgY or B cells expressing IgY [77,78,79]. This may be a key modulation in avian immune systems, supporting the evident B lymphocyte-mediated response to SA observed in the present study.

### 4.4. Primary and Recall Systemic Antibody Responses Against Intradermal Staphylococcus Aureus Injections

Primary and recall antibody responses to i.d. (GF pulp) killed SA vaccines (Figure 9) showed similar trends in IgM, IgY, and IgA profiles over time across all treatment groups. Specifically, the primary i.d. injections of the present study resulted in maximal SA-specific IgM levels in blood plasma by 7 d PV, which remained elevated throughout 28 d PV, similar to the IgM responses to i.d. injections of Salmonella-killed vaccines into GF pulps of chickens, as reported by Santamaria et al. [35] and Beck et al. [36]. Moreover, the dynamics of primary and recall IgY profiles in response to i.d. SA vaccines closely resemble the trend of the respective SA-specific IgM response, reaching highest levels at 7 d and 10 d PV, respectively, but returning to lower levels by 28 d, followed by gradually increasing IgA levels from 0 h through 21 d PV. This reflects characteristics of a T lymphocyte-mediated humoral response, with an isotype switch from SA-specific IgM to IgG and IgA, as explained by Santamaria et al. [32] in broilers i.d. vaccinated with formalin-killed Salmonella Enteritidis (SE). In their study, they observed maximal levels of SE-specific plasma IgM at 7 d, which then declined gradually (with IgM remaining well above baseline by 28 d). SE-specific IgY increased gradually from 0 d to 30 d PV. In contrast, SE-specific IgA reached maximal levels at 10 d and remained above pre-injection levels thereafter. Plasma IgA levels also remained relatively low in both primary and recall responses across all treatments compared to IgM and IgY, similar to the response to i.d. Salmonella-killed vaccines in the above studies [35,36]. One explanation is that peripheral blood may not reflect the dynamics of specific IgA profiles as well as mucus. Perera et al., 2025 [17] showed increased mucosal IgA profiles in broiler chickens injected with eBeam-killed multi-strain *Staphylococcus* compared with sham and FK groups. Still, these trends are not reflected in their plasma IgA profiles. Conversely, McElhaney 2009 [80] illustrates the drawback of subcutaneous and intramuscular injections for eliciting robust mucosal immunity. This may explain the early (D7 PV) significant increase in circulating IgA in the eB and FK groups of *in ovo* vaccinated broilers (Figure 2), but not in the i.d.-injected broilers. Additionally, towards the end of the primary response (14 d to 28 d PV), SA-specific IgM and IgY profiles are elevated in the eB and FK groups, reflecting the characteristics of a classic immune response. However, at 7 d and 10 d PV of the primary response, and throughout the recall response, SA-specific IgM and IgY profiles in the sham groups remain elevated, for which the reason is unclear. Similar dynamics of higher baseline IgY levels in PBS-vaccinated broilers, compared with similar or lower IgY levels in broilers immunized i.d. or subcutaneously with mannosylated chitosan-based or CD40-agonistic DNA aptamer-based inactivated Salmonella Enteritidis vaccines at 7 d PV, have also been reported [81].

## 5. Conclusions

This study is the first longitudinal investigation of anti-*Staphylococcus aureus* local and systemic immune responses in broiler chickens, including evaluation of primary and recall responses to eBeam- and formalin-killed SA vaccines. Following *in ovo* vaccination, broilers in the eBeam vaccine group generated significantly higher SA-specific IgM and IgA profiles in the blood early on than the control group, indicating superior protection at mucosal surfaces. Both eB and FK vaccines elicited equally high levels of total lymphocytes and B cells in the blood by the end of the first week of hatch. Leukocyte responses in vaccinated broilers following *in ovo* injections were mainly lymphocyte-driven, with higher total lymphocyte presence than heterophils and monocytes in circulation compared to sham. The eBeam vaccine group dominated the systemic total lymphocyte response for both modes of administration (*in ovo* or intradermal), generating the highest blood total lymphocyte concentrations compared with the formalin and sham vaccine groups. Cumulative effects of superior humoral and cell-mediated immunity illustrate the eBeam vaccine’s comparative immunogenicity across a broad spectrum following *in ovo* administration.

Considering local responses, the eBeam vaccine elicited the highest presence of heterophils and macrophages at 6 h post-vaccination during the primary immune response following intradermal injections into GF pulps, thereby generating a robust local inflammatory response. Additionally, the eBeam vaccine generated the highest B cell levels in circulation during both primary and recall responses to intradermal injections, followed by elevated B cell levels in GF pulps, on par with those of the formalin vaccine. Altogether, the eBeam vaccine generated superior B lymphocyte-mediated immune responses irrespective of the mode of administration. The local T-lymphocyte-mediated responses elicited by both eBeam and formalin vaccines were comparable, with prominent primary and recall CD4^+^ T cell responses relative to CD8^+^ T cell activity, indicating increased priming of T helper cell activity. All local lymphocyte responses within GF pulps showed higher peak lymphocyte levels in both the eB and FK vaccine groups during the recall response compared to the primary response, indicating that both vaccines successfully generated immunological memory against *Staphylococcus aureus*.

## Figures and Tables

**Table 1:** Treatment design of the experiment.

Group	Sub-Group	Total Number of Birds	*In Ovo* Vaccine Received ^1^	Intradermal Vaccine Received ^2^
A	A-EB	35	eBeam ^3^	eBeam ^3^
	A-FK	35	Formalin ^4^	Formalin ^4^
	A-Sham	35	Sham ^5^	Sham ^5^
B	B-EB	7	Sham ^5^	eBeam ^3^
	B-FK	7	Sham ^5^	Formalin ^4^
	B-Sham	7	Sham ^5^	Sham ^5^

^1^ A total of 100 µL of vaccine was administered into the amniotic sac of the embryo at day 18 of incubation. ^2^ A total of 10 µL of vaccine was administered into 22 growing feather pulps/bird on day 35 post-hatch. ^3^ Electron beam-killed *S. aureus* at a concentration of 1 × 10^7^ CFU/mL in endotoxin-free PBS. ^4^ Formalin-killed *S. aureus* at a concentration of 1 × 10^7^ CFU/mL in endotoxin-free PBS. ^5^ Endotoxin-free PBS without bacteria.

**Table 2:** Combinations of immunofluorescence-conjugated antibodies for immunofluorescent staining of leukocytes in growing feather pulp cell suspensions and whole blood cell suspensions.

Phase ^1^ of the Experiment and the Sample Stained	Stain Combination	Mac-Antibody ^2^	Label ^3^	Detected Leukocyte
Phase 1—blood	A	CD41/61	FITC	Thrombocytes
		CD45	SPRD	All leukocytes
	B	CD4	FITC	CD4^+^ T lymphocytes
		TCRγδ	PE	γδ T lymphocytes
		CD8α	SPRD	CD8α^+^ T lymphocytes
	C	KUL01	FITC	Monocytes
		Bu-1	PE	B lymphocytes
		CD3	SPRD	All T lymphocytes
Phase 2—blood	A	CD41/61	FITC	Thrombocytes
		KUL01	PE	Monocytes
		CD45	SPRD	All leukocytes
	B	CD4	FITC	CD4^+^ T lymphocytes
		TCRγδ	PE	γδ T lymphocytes
		CD8α	SPRD	CD8α^+^ T lymphocytes
	C	Bu-1	PE	B lymphocytes
		CD3	SPRD	All T lymphocytes
Phase 2—growing feather pulp suspensions	A	KUL01	PE	Macrophages
		CD45	SPRD	All leukocytes
	B	TCRγδ	FITC	γδ T lymphocytes
		CD4	PE	CD4^+^ T lymphocytes
		CD8α	SPRD	CD8α^+^ T lymphocytes
	C	TCRαβ	FITC	αβ T lymphocytes
		Bu-1	PE	B lymphocytes
		CD3	SPRD	All T lymphocytes

^1^ Phase 1—samples collected from the point of *in ovo* vaccination to the point of pre-growing feather (GF)-pulp injection (primary reaction of broilers), and phase 2—samples collected from the point of GF-pulp intradermal injections until the end of the experiment (primary and recall reactions of broilers). ^2^ mac—mouse anti-chicken monoclonal antibodies (Southern Biotechnology Associates, Inc., Birmingham, AL, USA). All antibodies were used at a 1:100 dilution. As antibodies for chicken heterophil identification are not available, this population was identified by side-scatter characteristics of cells in the CD45^+^-pulp cell suspension or the CD45^+^CD41/61^−^ blood cell suspensions. ^3^ FITC—fluorescein isothiocyanate, PE—phycoerythrin, SPRD—SpectralRed^TM^ conjugate.

## Data Availability

The raw data supporting the conclusions of this article will be made available by the authors on request.
